# Alzheimer-associated Aβ oligomers impact the central nervous system to induce peripheral metabolic deregulation

**DOI:** 10.15252/emmm.201404183

**Published:** 2015-01-23

**Authors:** Julia R Clarke, Natalia M Lyra e Silva, Claudia P Figueiredo, Rudimar L Frozza, Jose H Ledo, Danielle Beckman, Carlos K Katashima, Daniela Razolli, Bruno M Carvalho, Renata Frazão, Marina A Silveira, Felipe C Ribeiro, Theresa R Bomfim, Fernanda S Neves, William L Klein, Rodrigo Medeiros, Frank M LaFerla, Jose B Carvalheira, Mario J Saad, Douglas P Munoz, Licio A Velloso, Sergio T Ferreira, Fernanda G De Felice

**Affiliations:** 1 Institute of Medical Biochemistry Leopoldo de Meis, Federal University of Rio de JaneiroRio de Janeiro, RJ, Brazil; 2 School of Pharmacy, Federal University of Rio de JaneiroRio de Janeiro, RJ, Brazil; 3 Department of Internal Medicine, Faculty of Medical Sciences, State University of CampinasCampinas, SP, Brazil; 4 Department of Anatomy, Institute of Biomedical Sciences, University of São PauloSP, Brazil; 5 Department of Neurobiology, Northwestern UniversityEvanston, IL, USA; 6 Institute for Memory Impairments and Neurological Disorders, University of CaliforniaIrvine, CA, USA; 7 Center for Neuroscience Studies, Queen's UniversityKingston, ON, Canada; 8 Institute of Biophysics Carlos Chagas Filho, Federal University of Rio de JaneiroRio de Janeiro, RJ, Brazil

**Keywords:** Alzheimer's disease, ER stress, hypothalamus, inflammation, insulin resistance

## Abstract

Alzheimer's disease (AD) is associated with peripheral metabolic disorders. Clinical/epidemiological data indicate increased risk of diabetes in AD patients. Here, we show that intracerebroventricular infusion of AD-associated Aβ oligomers (AβOs) in mice triggered peripheral glucose intolerance, a phenomenon further verified in two transgenic mouse models of AD. Systemically injected AβOs failed to induce glucose intolerance, suggesting AβOs target brain regions involved in peripheral metabolic control. Accordingly, we show that AβOs affected hypothalamic neurons in culture, inducing eukaryotic translation initiation factor 2α phosphorylation (eIF2α-P). AβOs further induced eIF2α-P and activated pro-inflammatory IKKβ/NF-κB signaling in the hypothalamus of mice and macaques. AβOs failed to trigger peripheral glucose intolerance in tumor necrosis factor-α (TNF-α) receptor 1 knockout mice. Pharmacological inhibition of brain inflammation and endoplasmic reticulum stress prevented glucose intolerance in mice, indicating that AβOs act via a central route to affect peripheral glucose homeostasis. While the hypothalamus has been largely ignored in the AD field, our findings indicate that AβOs affect this brain region and reveal novel shared molecular mechanisms between hypothalamic dysfunction in metabolic disorders and AD.

## Introduction

Increasing evidence suggests an association between metabolic disorders, notably type 2 diabetes (T2D), and Alzheimer's disease (AD) (Craft, 2007; De Felice, 2013). Clinical and epidemiological studies indicate that diabetic patients have increased risk of developing AD (Ott *et al*, 1999; Sims-Robinson *et al*, 2010; Wang *et al*, 2012) and AD brains exhibit defective insulin signaling (Moloney *et al*, 2010; Bomfim *et al*, 2012; Craft, 2012; Talbot *et al*, 2012). Recent studies have shown that soluble amyloid-β peptide oligomers (AβOs), toxins that build up in AD brains and have been proposed to be major players in synapse failure in AD (reviewed in Ferreira & Klein, 2011; Selkoe, 2011; Mucke & Selkoe, 2012), are linked to impaired hippocampal insulin signaling. AβOs were found to cause internalization and cellular redistribution of insulin receptors, to block downstream hippocampal insulin signaling (De Felice *et al*, 2009; Ma *et al*, 2009; Bomfim *et al*, 2012) and to cause hippocampal endoplasmic reticulum (ER) stress (Lourenco *et al*, 2013), establishing molecular parallels between AD and T2D. Hyperinsulinemic/hyperglycemic individuals and mice show increased plasma and brain levels of Aβ (Ho *et al*, 2004; Takeda *et al*, 2010; Zhang *et al*, 2012), suggesting that altered peripheral metabolic homeostasis may increase Aβ levels and influence AD development (De Felice, 2013; De Felice & Ferreira, 2014).

Intriguingly, AD has been associated with increased risk of T2D development (Janson *et al*, 2004), suggesting that the connection between AD and T2D may be a two-way road. Early studies demonstrated peripheral glucose intolerance in AD patients (Craft *et al*, 1992). Recently, hyperglycemia and hyperinsulinemia, cardinal features of T2D and other metabolic disorders, were found to positively correlate with the development of AD-like brain pathology in humans (Matsuzaki *et al*, 2010). Obesity-induced insulin resistance is exacerbated in transgenic mouse models of AD (Takeda *et al*, 2010; Jimenez-Palomares *et al*, 2012). However, the molecular mechanisms underlying these observations are still largely unknown.

We hypothesized that AβOs could impact brain regions responsible for metabolic control and therefore represent a key pathogenic link between AD and deregulated peripheral glucose homeostasis. The hypothalamus plays a central role in neuroendocrine interaction between the central nervous system and the periphery (Schwartz & Porte, 2005; Koch *et al*, 2008). Emerging evidence further indicates that hypothalamic inflammation and ER stress are critical pathogenic events in the establishment of peripheral insulin resistance in metabolic disorders (Zhang *et al*, 2008; Milanski *et al*, 2009; Denis *et al*, 2010; Arruda *et al*, 2011; Thaler *et al*, 2012). An interesting recent study showed that hypothalamic inflammation accelerates aging and shortens lifespan in mice (Zhang *et al*, 2013). In *postmortem* AD brains, early studies identified Aβ deposits in the hypothalamus (Ogomori *et al*, 1989; Standaert *et al*, 1991). More recently, voxel-based morphometry revealed reduced hypothalamic volume in early AD compared to healthy controls (Loskutova *et al*, 2010), and a decrease in the number of hypothalamic orexin neurons has been reported in AD brains (Fronczek *et al*, 2012). In rats that received an intracerebroventricular injection of amyloid-β_25–35_ fibrils, Zussy *et al*, (2011) detected accumulation of fibrillar aggregates in the hypothalamus for as long as 3 weeks after the injection, as well as hypothalamic astrocytosis. In addition, oligomeric species of the amyloid-β peptide were recently shown to induce oxidative stress in a hypothalamic cell line (Gomes *et al*, 2014). While the hypothalamus has been largely ignored in the AD field, these studies indicate that this brain region could indeed be affected in AD. If so, hypothalamic dysfunction may have important consequences, predisposing AD patients to develop diabetes.

Several studies have established that AβOs target hippocampal neurons and induce synapse loss and neuronal dysfunction, eventually leading to memory impairment in AD (Ferreira & Klein, 2011; Mucke & Selkoe, 2012; Selkoe, 2012). Intracerebroventricular (i.c.v.) administration of AβOs has been shown to cause synapse loss and behavioral alterations linked to AD in mice (Figueiredo *et al*, 2013; Ledo *et al*, 2013) and AD-like pathology in non-human primates (Forny-Germano *et al*, 2014), providing a suitable model to investigate mechanisms germane to AD. Here, we show that i.c.v.-injected AβOs induce peripheral glucose intolerance and hallmarks of insulin resistance, including adipose tissue inflammation and impaired insulin-induced surface translocation of GLUT-4 in skeletal muscle. Peripheral glucose intolerance appeared to be mediated by a direct effect of AβOs in the central nervous system, and not by leakage of oligomers to peripheral tissues, as peripherally administered AβOs failed to induce glucose intolerance in mice. Glucose intolerance was further verified in two transgenic mouse models of AD, namely 3xTg-AD (Oddo *et al*, 2003) and APP/PS1 (Jankowsky *et al*, 2001) mice. We show that AβOs target primary hypothalamic neurons *in vitro* and accumulate in the hypothalamus of cynomolgus macaques given i.c.v. infusions of AβOs. AβOs further triggered aberrant generation of reactive oxygen species (ROS) and phosphorylation of eIF2α in cultured hypothalamic neurons, as well as activation of IKKβ/NF-κB inflammatory signaling in the hypothalamus of mice and macaques. The impact of AβOs in the hypothalamus of mice preceded alterations in peripheral glucose homeostasis. In TNF-α receptor 1 knockout mice (Romanatto *et al*, 2009), AβOs failed to trigger hypothalamic IKK activation and IRS-1 inhibition. AβO-associated glucose intolerance was prevented in TNFR1^−/−^ mice as well as in wild-type mice given i.c.v. infusions of tauroursodeoxycholic acid (TUDCA), an ER stress inhibitor. i.c.v treatment with infliximab, a TNF-α neutralizing antibody, further prevented glucose intolerance in AβO-injected mice and in APP/PS1 mice. Collectively, results establish a novel pathogenic mechanism by which AβOs impact the hypothalamus, causing peripheral metabolic deregulation.

## Results

### Mouse models of AD exhibit impaired glucose tolerance

**Figure 1:**
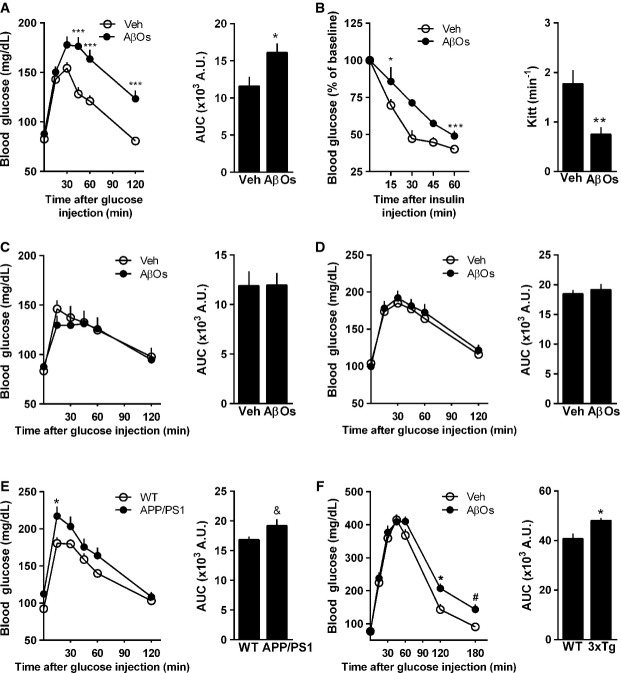
AD mouse models show peripheral glucose intolerance Adult Swiss mice (*n *=* *11 Veh; 15 AβOs) received a single i.c.v. injection of vehicle or 10 pmol AβOs and were assessed in a glucose tolerance test (2 g glucose/kg body weight, i.p.) 7 days after injection. Blood levels of glucose were measured at several time points following glucose administration. Bar graph represents areas under the curves in the time course plot. Data are representative of three independent experiments with similar results. Left panel: ***P =* *0.0006, two-way ANOVA followed by Bonferroni *post hoc* test; right panel: *P =* *0.0207, Student's *t*-test.

Alzheimer's disease has been associated with increased risk of T2D development. We hypothesized that brain accumulation of AβOs could represent a key pathogenic link between AD and deregulated peripheral glucose homeostasis. To test this hypothesis, we initially performed a single injection of 10 pmol AβOs into the right lateral cerebral ventricle of adult Swiss mice (Supplementary Fig S1; Figueiredo *et al*, 2013; Ledo *et al*, 2013). AβOs were freshly prepared before each experiment and were routinely characterized by size-exclusion chromatography, Western blots using anti-oligomer monoclonal antibody NU4 (Lambert *et al*, 2007) and, occasionally, by transmission electron microscopy, as previously described (Jurgensen *et al*, 2011; Sebollela *et al*, 2012; Figueiredo *et al*, 2013). Interestingly, mice that received an i.c.v. injection of AβOs exhibited impaired peripheral glucose tolerance and insulin resistance 7 days after injection (Fig1A and B). Control experiments showed that peripheral glucose tolerance was unaffected by i.c.v. injection of a preparation of scrambled Aβ peptide submitted to the same oligomerization protocol used for regular AβO preparations (Supplementary Fig S2A). The impairment in glucose tolerance induced by i.c.v. AβOs was comparable to that verified in mice submitted to a high-fat diet for 7 days (Supplementary Fig S2B). Impaired glucose tolerance could be detected as early as 36 h, but not 12 h after i.c.v. injection of AβOs (Supplementary Fig S2D and C), and persisted for at least 14 days post-injection (Supplementary Fig S2E). We further examined the possibility that leakage of AβOs from the brain might explain the observed effects on peripheral glucose metabolism. To this end, we injected 10 pmol AβOs (the same amount used in i.c.v. injections) directly into the caudal vein or into the peritoneum of mice. In either case, systemic administration of AβOs failed to impair glucose tolerance (Fig1C and D), ruling out a direct action of AβOs on peripheral tissues in our conditions.

Insulin tolerance test (1 IU insulin/kg body weight, i.p.) (*n *=* *7 Veh; 8 AβOs). Blood levels of glucose were measured at several time points following insulin administration. Bar graph represents the kinetic constants for glucose disappearance (Kitt) calculated from the time course plot. Data are representative of two independent experiments with similar results. Left panel: *P =* *0.0456 and ***P =* *0.0007, two-way ANOVA followed by Bonferroni *post hoc* test; right panel: **P =* *0.0033, Student's *t*-test.

Glucose tolerance test (2 g glucose/kg body weight, i.p.) in mice 7 days after a single intracaudal (C; *n *=* *8 animals/group) or intraperitoneal (D; *n *=* *13 animals/group) injection of AβOs (10 pmol) or vehicle.

Glucose tolerance test (2 g glucose/kg body weight, i.p.) in 8- to 13-month-old APP/PS1 mice (E; *n *=* *9 animals/group) or 6-month-old 3xTg-AD male mice (F; *n *=* *10 WT; 9 3xTg), or their corresponding wild-type littermates. Bar graph represents areas under the curves in the time course plots. In (E), left panel: *P =* *0.0466, two-way ANOVA followed by Bonferroni *post hoc* test; right panel: ^&^*P *=* *0.072, Student's *t*-test. In (F), left panel: *P =* *0.0171 and ^#^*P *=* *0.0781, two-way ANOVA followed by Bonferroni *post hoc* test; right panel: *P =* *0.0101, Student's *t*-test. Data information: Data are expressed as means ± SEM.

Significantly, altered peripheral glucose homeostasis was also verified in 9- to 13-month-old APPSwePS1ΔE9 (APP/PS1) mice compared to wild-type animals (Fig1E). Those mice harbor transgenes for human amyloid precursor protein (APP) bearing the Swedish mutation and a deletion mutant form of presenilin 1 (Shi *et al*, 2011a), and present increased Aβ production and cognitive deficits (Jankowsky *et al*, 2001). Similar results were obtained using the triple-transgenic mouse model of AD (3xTg-AD), which presents increased Aβ levels and develops tau and synaptic pathology, hallmark features of AD (Oddo *et al*, 2003). We found that 6-month-old 3xTg-AD mice show glucose intolerance compared to wild-type littermates (Fig1F). The fact that altered peripheral glucose homeostasis was detected in both mouse models exhibiting progressive Aβ accumulation in the brain underscores the notion that our observations in the acute model consisting of brain infusion of AβOs are relevant when compared to clinical observations in early AD patients (Craft *et al*, 1992).

### i.c.v. injection of AβOs induces metabolic changes in muscle and adipose tissue and increases plasma noradrenaline levels

**Figure 2:**
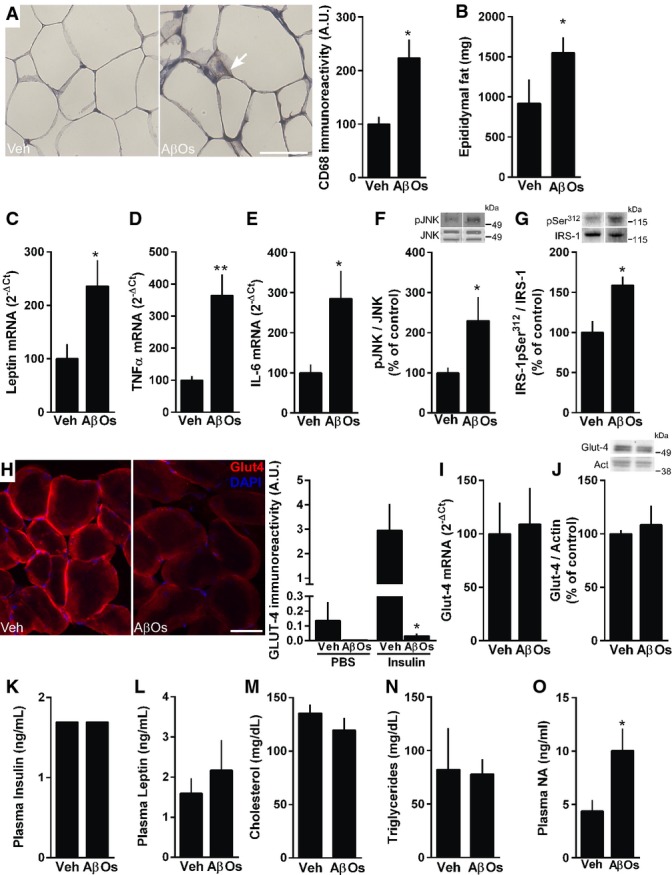
i.c.v-injected AβOs induce adipose tissue inflammation and insulin resistance in muscle CD68 immunoreactivity in white adipose tissue (scale bar = 25 μm, images representative of one animal each from a total of four animals per experimental group). Arrow points to a region stained with CD68 antibody. *P =* *0.0109, Student's *t*-test.

We next sought to analyze metabolic changes and insulin responsiveness in metabolically active tissues. We found increased CD68 immunoreactivity in adipose tissue of mice that received an i.c.v. injection of AβOs (Fig2A), indicating macrophage/myeloid cell infiltration. Further, AβO-injected mice had higher amounts of epididymal fat (Fig2B) and increased expression of leptin and pro-inflammatory cytokines, TNF-α and IL-6, in white adipose tissue (Fig2C–E). In obese mice, adipose-derived TNF-α is involved in insulin resistance through the activation of JNK, leading to increased inhibitory serine phosphorylation of insulin receptor substrate-1 (IRS-1pSer) in muscle (Hotamisligil *et al*, 1996; Ozcan *et al*, 2004). Therefore, we investigated whether this pathway was affected in AβO-injected mice. Indeed, skeletal muscle from mice i.c.v. injected with AβOs showed increased levels of activated JNK (Fig2F) and IRS-1pSer^312^ (Fig2G). Physiologically, insulin signaling in muscle induces translocation of glucose transporter-4 (GLUT-4) from intracellular compartments to the plasma membrane (Huang & Czech, 2007). In line with our finding of IRS-1 inhibition, insulin-stimulated translocation of GLUT-4 to the plasma membrane was severely impaired in skeletal muscle of mice that received an i.c.v. injection of AβOs (Fig2H), while GLUT-4 expression and total protein levels in muscle remained unaltered (Fig2I and J).

Epididymal fat mass was analyzed in mice (*n *=* *6 animals/group) 7 days after i.c.v. injection of vehicle or AβOs. Data are representative of three independent experiments with similar results. *P =* *0.0255.

Relative expression of leptin (C), TNF-α (D) and IL-6 (E), respectively, in white adipose tissue of mice (*n *=* *7 Veh; 9 AβOs) 7 days after i.c.v. injection of vehicle or AβOs. In (C), *P =* *0.0394; in (D), **P =* *0.0038; in (E), *P =* *0.0305; Student's *t*-test.

p-JNK (F; *n *=* *5 animals/group) and IRS-1pSer^312^ (G; *n *=* *6 animals/group) levels (normalized by total JNK and total IRS-1, respectively) in skeletal muscle of mice 7 days after i.c.v. injection of vehicle or AβOs. In (F), *P =* *0.0464; in (G), *P =* *0.0081; Student's *t*-test.

Representative images of GLUT-4 immunofluorescence in insulin-stimulated skeletal muscle from mice that were i.c.v.-injected with vehicle (Veh) or 10 pmol AβOs. Bar graphs show quantification of GLUT-4 surface immunoreactivity in skeletal muscle of mice that received intraperitoneal injections of PBS or insulin (1 IU/kg body weight) 7 days after i.c.v. injection of vehicle or AβOs, as indicated (*n *=* *5 animals/group). Scale bar = 25 μm. *P =* *0.0144, one-way ANOVA followed by Bonferroni *post hoc* test.

GLUT-4 mRNA (*n *=* *4 animals/group) and total protein levels (normalized to actin levels; *n *=* *5 Veh; 6 AβOs) were unchanged in skeletal muscle of Swiss mice injected with vehicle (Veh) or 10 pmol AβOs.

Plasma levels of insulin (K; *n *=* *12 animals/group), leptin (L; *n *=* *11 Veh; 12 AβOs), cholesterol (M; *n *=* *8 Veh; 6 AβOs), triglycerides (N; *n *=* *8 Veh; 6 AβOs) or noradrenaline (O; *n *=* *7 Veh; 8 AβOs) measured 7 days after i.c.v. injection of vehicle (Veh) or 10 pmol AβOs. In (O), *P =* *0.0361, Student's *t*-test. Data information: Data are expressed as means ± SEM, and data are representative of two independent experiments with similar results. To assess statistical significance, AβO-injected mice were compared to vehicle-injected mice. Source data are available online for this figure.

In order to provide a more comprehensive view of metabolic deregulation in AβO-injected mice, we next measured serum levels of leptin and insulin in mice 7 days after i.c.v. injection of AβOs. We found no changes in serum levels of insulin or leptin under these conditions (Fig2K and L). As noted above, the impairment in glucose tolerance induced by i.c.v. administration of AβOs is comparable to that verified in mice submitted to a high-fat diet (HFD) for 7 days (Supplementary Fig S2B). In harmony with our results, previous studies have shown that plasma leptin and insulin levels are not affected in mice (wild-type or ob/ob) submitted to a short-term (4–7 days) high-fat diet (HFD), whereas glucose tolerance and insulin sensitivity are clearly impaired under the same conditions (e.g., El-Haschimi *et al*, 2000; Ji *et al*, 2012; Le *et al*, 2014). Further, short-term HFD induces increases in epididymal white adipose tissue weight, adipocyte hypertrophy and increased transcript levels of TNF-α and IL-6 (e.g., Lee *et al*, 2011; Ji *et al*, 2012), similar to our observations in mice i.c.v. injected with AβOs. Moreover, plasma levels of cholesterol and triglycerides were comparable between vehicle- and AβO-injected animals (Fig2M and N). We further found elevated plasma noradrenaline (NA) levels (Fig2O), indicating that AβOs cause deregulation of peripheral sympathetic control.

### AβOs bind to hypothalamic neurons in culture and induce aberrant ROS generation and TNF-α-dependent increase in eIF2α-P

**Figure 3:**
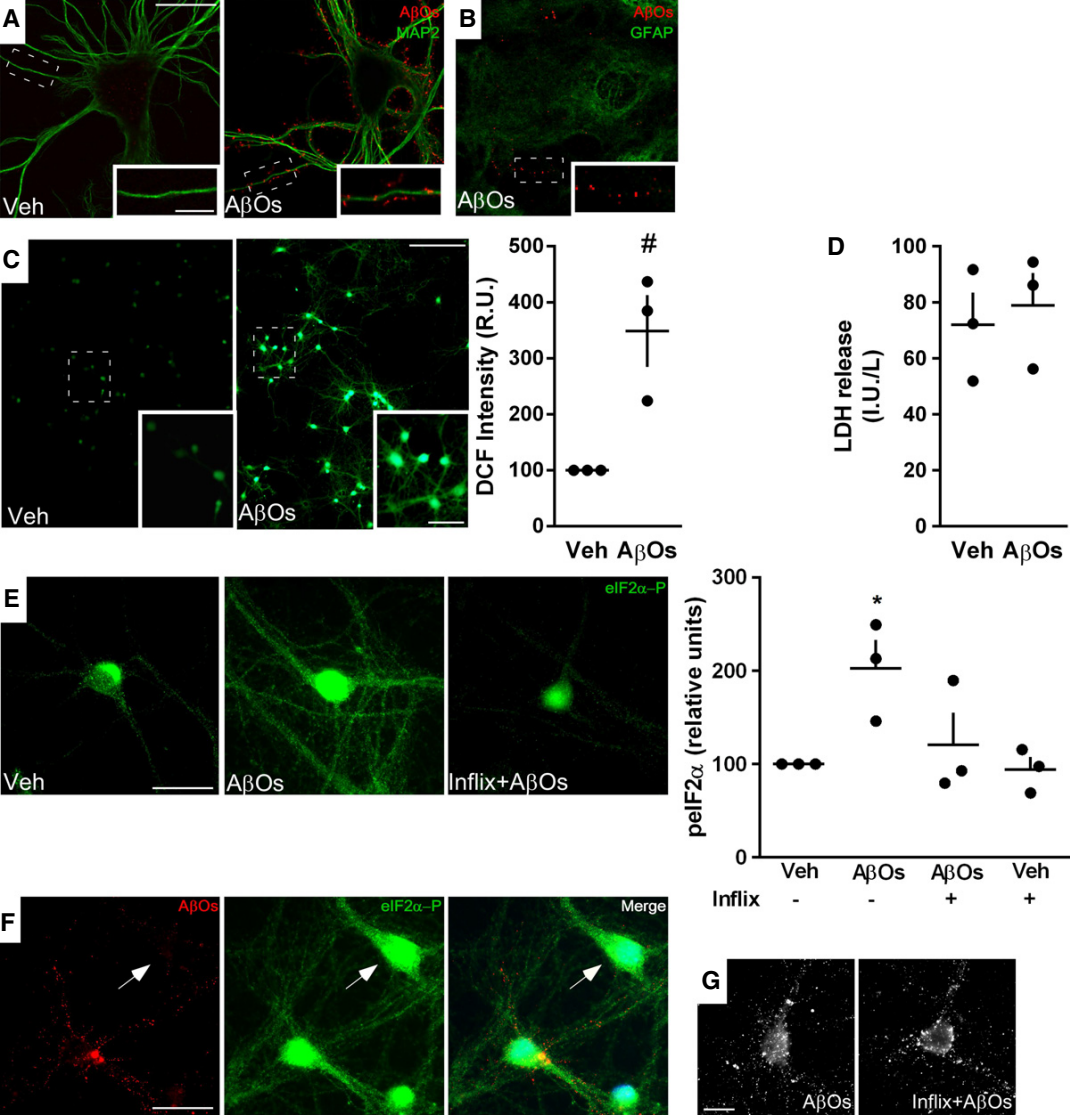
AβOs bind to and impact hypothalamic neurons Representative immunocytochemistry images of mature hypothalamic neurons in culture exposed to vehicle (Veh) or AβOs (500 nM) for 3 h. Binding of AβOs was detected using anti-oligomer monoclonal antibody NU4 (red). Neurons were double-labeled using MAP-2 antibody (green). Images represent typical results from experiments with three independent hypothalamic cultures (three coverslips/experimental condition per independent experiment). Scale bar = 30 and 10 μm for main panels and insets, respectively.

Since i.p. or i.v. administration of AβOs had no effect on peripheral glucose homeostasis, we hypothesized that AβOs could target brain regions involved in control of peripheral glucose homeostasis. Because interference in the hypothalamus of mice has been shown to be sufficient to induce peripheral metabolic deregulation (Purkayastha *et al*, 2011), and early studies showed that Aβ accumulates in the hypothalamus of AD patients, we next aimed to determine whether this brain region was particularly affected in our experimental models. Initially, highly differentiated primary hypothalamic neuronal cultures were exposed to AβOs (500 nM) for 3 h and AβO binding to neurons was investigated by double immunofluorescence labeling using oligomer-sensitive antibody NU4 (Lambert *et al*, 2007) and microtubule-associated protein 2 (MAP-2). Results showed that AβOs bind to the soma and, especially, to dendrites of selected hypothalamic neurons (Fig3A), similar to previous results demonstrating that oligomers bind to a specific subset of neurons in hippocampal cultures, rather than to all neurons (Lacor *et al*, 2004; Zhao *et al*, 2008; Bomfim *et al*, 2012; Lourenco *et al*, 2013). To examine the possibility that AβOs could bind to astrocytes, we further double-labeled cultures with anti-GFAP and NU4. Results indicate that oligomers do not bind to astrocytes in culture (Fig3B). We further asked whether AβOs would instigate oxidative stress in primary hypothalamic neurons in culture, as previously shown in hippocampal neurons (De Felice *et al*, 2007) and in a hypothalamic cell line (Gomes *et al*, 2014). We found that AβOs induce a robust increase in reactive oxygen species (ROS) levels in cultured hypothalamic neurons (Fig3C). Under the same conditions, the lactate dehydrogenase cytotoxicity assay provided no evidence of cell death induced by exposure to AβOs in culture (Fig3D).

Representative immunocytochemistry image of mature hypothalamic culture exposed to AβOs (500 nM) and immunolabeled with anti-GFAP (green) and NU4 (red) antibodies. Insets show AβOs binding to neuronal dendrites, whereas no binding was detected to GFAP-positive cells.

Representative DCF fluorescence images from hypothalamic neuronal cultures exposed to vehicle or AβOs (500 nM, 4 h). Insets show optical zoom images of the indicated areas. Scale bars = 100 and 50 μm for main panels and insets, respectively. Graph shows integrated DCF fluorescence intensities (relative units; see Materials and Methods) (*n *=* *3 independent hypothalamic cultures; three wells/experimental condition per experiment; three images acquired per well). Bars represent means ± SEM. ^#^*P *=* *0.0604, one-sample *t*-test compared with a fixed value of 100 RUs.

LDH activity (IU/l) in culture media of hypothalamic cultures exposed to vehicle or AβOs (500 nM, 3 h).

Representative immunofluorescence images of eIF2α-P in hypothalamic cultures exposed to vehicle or AβOs (500 nM, 3 h) in the absence or presence of infliximab (1 μg/ml). Scale bar = 30 μm. Graph represents integrated immunofluorescence intensities of eIF2α-P levels from three independent hypothalamic cultures (three coverslips/experimental condition per experiment, 20 images per coverslip). Bars represent means ± SEM. *P =* *0.0489, one-way ANOVA followed by Bonferroni *post hoc* test comparing AβO-treated versus vehicle-treated cultures.

Representative images of hypothalamic cultures exposed to AβOs (500 nM, 3 h) and double-labeled with NU4 (oligomer-sensitive) and eIF2α-P antibodies. Arrow points to a neuron presenting high levels of eIF2α-P in the absence of AβO binding. Nuclear staining (DAPI) is shown in blue. Scale bar = 30 μm.

Representative images of hypothalamic neurons labeled with NU4 antibody exposed to AβOs (500 nM, 3 h) in the absence or presence of infliximab (1 μg/ml). Similar patterns of AβO binding were observed in both conditions. Scale bar = 20 μm.

Because phosphorylation of eIF2α-P, one of the branches of the unfolded protein response (UPR) activated upon ER stress, was recently shown to underlie AβO toxicity in the hippocampus (Costa *et al*, 2012; Lourenco *et al*, 2013; Ma *et al*, 2013), and hypothalamic ER stress has been proposed to play an important role in the pathogenesis of metabolic disorders (Ozcan *et al*, 2004, 2006; Hotamisligil, 2010), we asked whether AβOs might trigger eIF2α-P in mature cultured hypothalamic neurons. We found increased eIF2α-pSer51 (eIF2α-P) in neuronal dendrites and cell bodies after exposure of neurons to AβOs for 3 h (Fig3E). Importantly, elevated eIF2α-P levels were found independent of whether or not neurons exhibited oligomers bound to their dendrites (Fig3F). This indicates that eIF2α phosphorylation is not triggered by direct binding of oligomers to individual neurons, but rather is instigated by soluble factors released to the medium upon exposure of cultures to AβOs. In a recent study, we found that pro-inflammatory TNF-α signaling induced eIF2α-P in hippocampal neurons (Lourenco *et al*, 2013). To determine whether TNF-α activation was involved in AβO-induced eIF2α-P in hypothalamic neurons, we treated cultures with infliximab, a TNF-α neutralizing monoclonal antibody. Infliximab attenuated eIF2α-P triggered by AβOs (Fig3E). It is noteworthy that infliximab did not block oligomer binding to neurons (Fig3G), substantiating the notion that activation of TNF-α/eIF2α-P signaling is independent of direct binding of AβOs to individual neurons and is likely mediated by TNF-α secreted to the medium.

### i.c.v. injection of AβOs induces increased hypothalamic inflammation and eIF2α-P in mice and macaques

**Figure 4:**
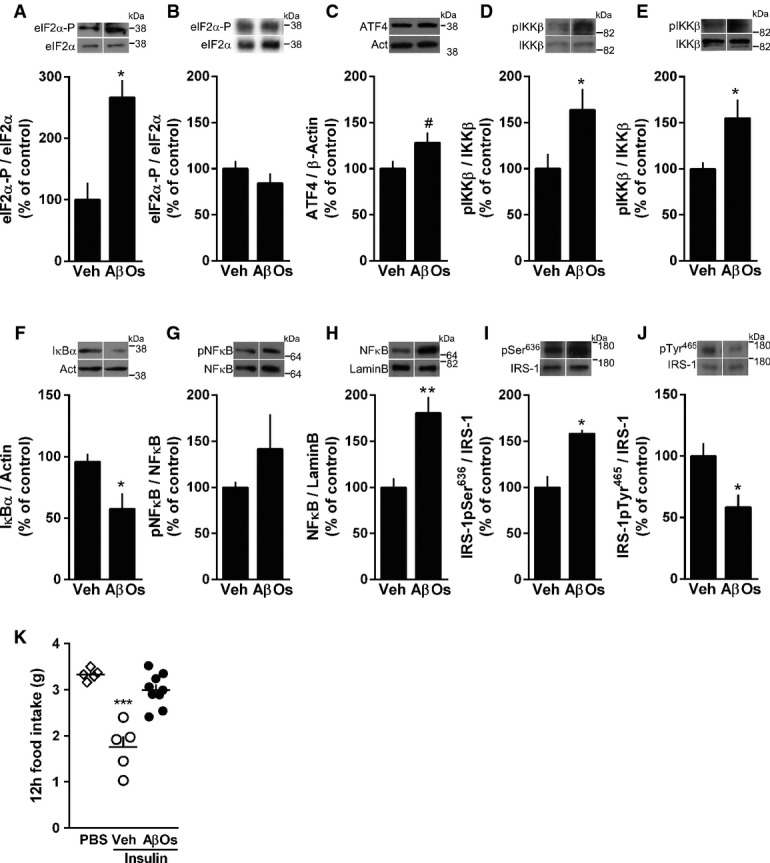
AβOs induce hypothalamic inflammation, eIF2α phosphorylation and impaired insulin signaling Western blot analysis of eIF2α-P levels in the hypothalamus of mice 4 h (A; *n *=* *4 animals/group) or 7 days (B; *n *=* *6 Veh; 5 AβOs) after a single i.c.v. injection of vehicle (Veh) or 10 pmol AβOs. Graphs show densitometric data normalized by total eIF2α levels. *P =* *0.0213.

We next asked whether i.c.v.-infused AβOs might trigger eIF2α-P in the mouse hypothalamus. We found a significant increase in hypothalamic levels of eIF2α-P 4 h after i.c.v. injection of AβOs (Fig4A), but not 7 days after oligomer injection (Fig4B). We next investigated levels of other components of the UPR 4 h after i.c.v. injection of AβOs. Consistent with increased eIF2α-P, levels of ATF4, a downstream effector of eIF2α, were increased in AβO-injected mice (Fig4C). Other ER stress markers analyzed remained unaltered, including PERKpThr980, ATF6, IRE1α-pSer724, spliced Xbp1 and Grp78 (Supplementary Fig S3A–G). We note that we have examined ER stress markers at a single time point (4 h post-AβO injection) and future studies aimed to analyze in more detail the time course of changes in levels of ER stress markers may provide additional insight into the mechanisms by which AβOs instigate hypothalamic deregulation.

Western blot analysis of ATF4 levels in the hypothalamus of mice 4 h after i.c.v. injection of vehicle (Veh) or 10 pmol AβOs; graph shows densitometric data normalized by β-actin (*n *=* *7 Veh; 8 AβOs). ^#^*P *=* *0.0731; Student's *t*-test.

Western blot analysis of hypothalamic phospho-IKKβ levels in the hypothalamus of mice 4 h (C; *n *=* *6 animals/group) or 7 days (D; *n *=* *4 Veh; 5 AβOs) after i.c.v. injection of vehicle or 10 pmol AβOs. Graphs show densitometric data normalized by total IKKβ levels. In (D), *P =* *0.0437; in (E), *P =* *0.0444; Student's *t*-test.

Western blot analysis of IκBα (F; *n *=* *6 animals/group) and cytoplasmic phospho-p65-NF-κB (G; *n *=* *4 Veh; 5 AβOs) in the hypothalamus of mice 4 h after i.c.v. injection of vehicle or 10 pmol AβOs. Graphs show densitometric data normalized by actin (F) or total NF-κB levels (G). *P =* *0.0207.

Nuclear NF-κB levels in the hypothalamus 6 h after i.c.v. injection of vehicle or 10 pmol AβOs in mice. Graphs show NF-κB levels normalized by nuclear marker, lamin B (*n *=* *6 animals/group). **P =* *0.0024; Student's *t*-test.

IRS-1pSer^636^ (I; *n *=* *4 animals/group) and pTyr^465^ (J; *n *=* *6 Veh; 4 AβOs) levels in the hypothalamus 7 days after i.c.v. injection of vehicle or AβOs in mice. Graphs show IRS-1pSer or IRS-1pTyr levels normalized by total IRS-1. In (I), *P =* *0.0043; in (J), *P =* *0.0275; Student's *t*-test.

Twelve-hour food intake after a single i.c.v. infusion of insulin (200 mU) in mice. Experiment was performed 7 days after i.c.v. injection of vehicle or AβOs (*n *=* *5 PBS; 5 Veh + Insulin; 9 AβOs + Insulin), data are representative of two independent experiments with similar results. ***P <* *0.0001, one-way ANOVA followed by Bonferroni *post hoc* test comparing Veh-Insulin versus PBS groups. Data information: Data are expressed as means ± SEM. In (A–J), to assess statistical significance, AβO-injected mice were compared to vehicle-injected mice. Source data are available online for this figure.

In animal models of T2D and obesity, an inflammatory response in the hypothalamus, notably via the activation of the IKKβ/NF-κB pathway, is an important part of the mechanism underlying pathogenesis (Zhang *et al*, 2008; Thaler *et al*, 2012). Compared to vehicle-injected mice, AβO-injected mice exhibited early activation of IKKβ in the hypothalamus (Fig4D, 4 h after i.c.v. injection), which persisted for 7 days after i.c.v. injection of AβOs (Fig4E). Once activated, IKKβ phosphorylates IκBα, which undergoes ubiquitination and proteasomal degradation, allowing NF-κB phosphorylation and nuclear translocation. Accordingly, we found decreased levels of IκBα (Fig4F), a trend of increase in cytoplasmic NF-κB phosphorylation (Fig4G), and significantly increased levels of NF-κB in the nucleus (Fig4H) in the hypothalamus of AβO-injected mice. On the other hand, no differences in activated JNK or PKR levels were detected in the hypothalamus of AβO-injected mice compared with vehicle-injected mice 4 h or 7 days after i.c.v. injection of oligomers (Supplementary Fig S4G–J).

We further found that IRS-1pSer^636^ levels were increased and IRS-1pTyr^465^ levels were decreased in the hypothalamus of mice 7 days after oligomer injection (Fig4I and J), indicating that AβOs impaired hypothalamic insulin signaling. To determine whether AβO-induced insulin resistance in neuroendocrine brain regions impaired the ability of the brain to respond to insulin signaling by reducing food intake, mice were kept in metabolic cages for 7 days following i.c.v. injection of AβOs (or vehicle) and food intake was measured following an acute i.c.v. infusion of insulin (Schwartz *et al*, 2000; Sanchez-Lasheras *et al*, 2010). Significantly, AβO-injected mice failed to exhibit the expected suppression in acute food intake upon i.c.v. administration of insulin, indicating central insulin resistance (Fig4K).

**Figure 5:**
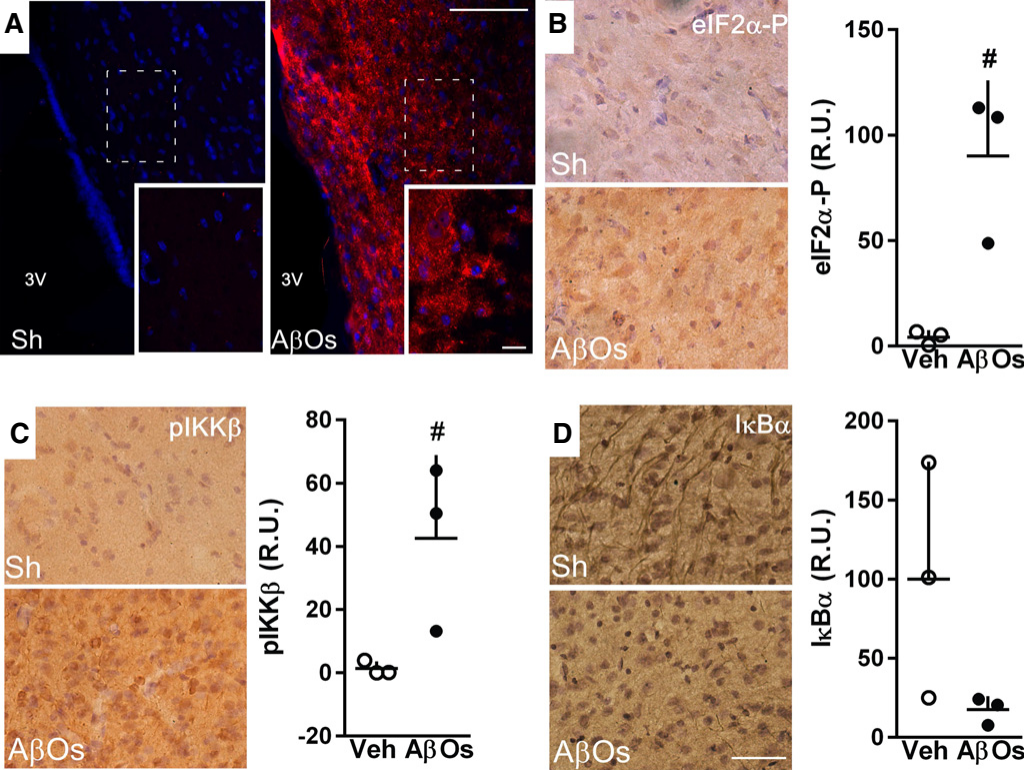
AβOs accumulate in the hypothalamus of macaques and induce inflammation and eIF2α phosphorylation Representative images of AβO immunoreactivity (detected using anti-oligomer monoclonal antibody NU4) in the hypothalamus of control, sham-operated adult cynomolgus macaques (Sh; *n *=* *3) or macaques that received i.c.v. injections of AβOs (*n *=* *3; see Materials and Methods). Nuclear staining (DAPI) is shown in blue. Insets show optical zoom images of the areas indicated by white dashed rectangles in the main panels. Scale bars = 100 and 20 μm for main panels and insets, respectively.

To determine the impact of AβOs in an animal model with greater proximity to humans, we have recently developed a non-human primate model of AD by delivering i.c.v. infusions of oligomers in adult cynomolgus macaques (Forny-Germano *et al*, 2014). Our previous studies showed that this macaque model of AD presents hippocampal IRS-1 pathology and elevated hipocampal eIF2α-P levels (Bomfim *et al*, 2012; Lourenco *et al*, 2013). Three macaques received i.c.v. injections of AβOs, while three sham-operated animals were used as controls, and their hypothalami were analyzed (Supplementary Fig S4). Strong AβO immunoreactivity was found in the hypothalamus of oligomer-injected macaques, but not in sham animals (Fig5A). We next investigated whether similar effects to those found in mice could be observed in AβO-injected macaques. We found significantly elevated hypothalamic levels of eIF2α-P (Fig5B) and pIKKβ (Fig5C), as well as a trend of decrease in hypothalamic IκBα levels in AβO-injected macaques (Fig5D). Results indicate that abnormal inflammatory signaling and ER stress are triggered by AβOs in the primate hypothalamus.

Representative images showing eIF2α-P (B), phospho-IKKβ (C) and IκBα (D) immunoreactivities in the hypothalamus of cynomolgus macaques that received i.c.v. injections of AβOs or control (sham-operated; Sh) macaques (*n *=* *3 animals/group). Graphs show immunolabeling optical density analysis from three images acquired in the hypothalamus of each macaque (three control versus three AβO-injected animals). In (B), ^#^*P *=* *0.0523; in (C) ^#^*P *=* *0.1123; unpaired Student's *t*-test with Welch's correction for unequal variances; AβO-injected monkeys compared to sham-operated monkeys. Scale bars = 50 μm

### AβOs induce increased expression of orexigenic peptides and chow intake in mice

**Figure 6:**
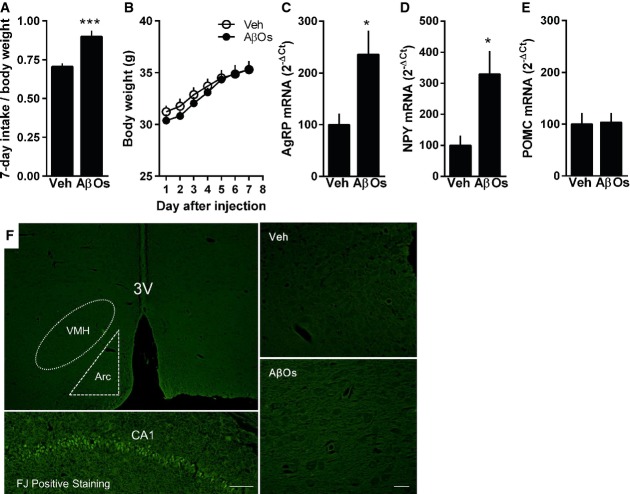
i.c.v.-injected AβOs induce increased food intake, hypothalamic expression of orexigenic neuropeptides but no hypothalamic cell degeneration Accumulated chow intake (normalized by body weight) measured during 7 days following a single i.c.v. injection of vehicle or 10 pmol AβOs in mice (*n *=* *13 Veh; 10 AβOs; data are representative of two independent experiments with similar results). ***P <* *0.0001; Student's *t*-test.

Intriguingly, AβO-injected mice presented increased chow intake (Fig6A), even though no significant differences in body weight (Fig6B) were found between experimental groups. Consistent with increased chow ingestion, elevated hypothalamic expression of orexigenic neuropeptides AgRP and NPY (but no alterations in anorexigenic POMC mRNA levels) was detected in AβO-injected mice (Fig6C–E). To gain insight into how AβOs cause the observed peripheral metabolic alterations, we asked whether AβO injection might lead to death of hypothalamic cells. We carried out Fluorojade staining in brain tissue from vehicle- or AβO-injected mice (7 days post-injection). Results showed no evidence of cell degeneration in AβO-injected mice compared to vehicle-injected animals (Fig6F). We next performed whole-cell patch-clamp recordings in brain slices from AβO-injected mice to determine whether AβOs affected hypothalamic neuron electrophysiology. We targeted cells from the arcuate nucleus, a region enriched in NPY neurons (Allen Brain Atlas [http://mouse.brain-map.org]; Hahn *et al*, 1998). No changes were detected in frequency or amplitude of either excitatory or inhibitory post-synaptic currents, or in resting membrane potential of the recorded neurons (Supplementary Fig S5A–E), suggesting that the mechanism by which AβOs induce functional deregulation of hypothalamic neurons does not include major alterations in their electrophysiological properties.

Daily body weight measured during 7 days after i.c.v. injection of vehicle or AβOs (*n *=* *7 animals/group; data are representative of two independent experiments with similar results).

Adult Swiss mice received a single i.c.v. injection of vehicle or 10 pmol AβOs, and hypothalamic levels of mRNA for AgRP (C; *n *=* *6 Veh; 5 AβOs), NPY (D; *n *=* *6 Veh; 5 AβOs) and POMC (E; *n *=* *7 animals/group) were analyzed 7 days after injection. In (C), *P =* *0.0191; in (D), *P =* *0.0115; Student's *t*-test.

Swiss mice received a single i.c.v. injection of vehicle (Veh) or 10 pmol AβOs, and their brains were analyzed by Fluorojade staining of degenerating cells 7 days after the injection. Representative images of Fluorojade staining in the hypothalamus of vehicle- or AβO-injected mice (*n *=* *4/group). Scale bar = 100 μm in left panels (top and bottom) and 20 μm in right panels (top and bottom). Positive control (bottom left panel) was the hippocampus of a mouse that received one i.c.v. injection of quinolinic acid (36.8 nmol) and was analyzed 24 h after. Data information: Data are expressed as means ± SEM. To assess statistical significance, AβO-injected mice were compared to vehicle-injected mice.

### Blockade of brain ER stress or inflammation attenuates glucose intolerance and normalizes plasma noradrenaline levels in mice

**Figure 7:**
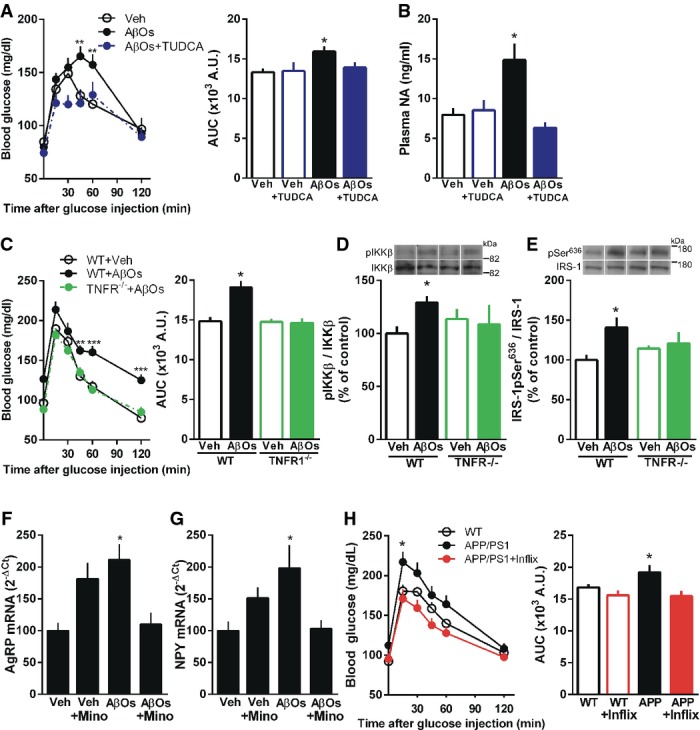
AβO-induced peripheral glucose intolerance and hypothalamic insulin resistance are mediated by TNF-α signaling and hypothalamic ER stress Glucose tolerance test (2 g glucose/kg body weight, i.p.) in mice that received i.c.v. injections of vehicle, vehicle + TUDCA, AβOs or AβOs + TUDCA (when used, TUDCA was administered in 5 i.c.v. injections of 5 μg TUDCA each, before and after oligomer injection; see Materials and Methods. Control groups received injections of saline). Glucose tolerance test (GTT) was performed 7 days after i.c.v. injection of vehicle or AβOs. Bar graph represents areas under the curves (AUC) in the time course plots (*n *=* *15 Veh; 15 AβOs; 10 Veh + TUDCA; 16 AβOs + TUDCA). Data are representative of two independent experiments with similar results. Left panel: **P =* *0.0048, **P =* *0.003, two-way ANOVA followed by Bonferroni *post hoc* test; right panel: *P =* *0.0384, one-way ANOVA followed by Bonferroni *post hoc* test.

Recent observations indicate that transient hypothalamic ER stress is sufficient to deregulate peripheral insulin signaling and upregulate peripheral sympathetic tonus (Purkayastha *et al*, 2011). Since we found that AβOs induce transient hypothalamic eIF2α-P (Fig4A and B) and increased plasma noradrenaline levels in mice (Fig2O), we next investigated whether prevention of brain ER stress could attenuate AβO-induced defects in peripheral glucose homeostasis and in plasma noradrenaline levels. We found that i.c.v. injections of tauroursodeoxycholic acid (TUDCA), a chemical chaperone that alleviates ER stress, prevented both the impairment in glucose tolerance and the increase in plasma noradrenaline levels induced by i.c.v.-injected AβOs (Fig7A and B). These results indicate that AβOs use a central route to cause deregulation of peripheral glucose homeostasis.

Plasma noradrenaline (NA) levels measured 7 days after i.c.v. injection of vehicle, vehicle + TUDCA, AβOs or AβOs + TUDCA in mice (*n *=* *7 animals/group). Data are representative of two independent experiments with similar results. *P =* *0.0071, one-way ANOVA followed by Bonferroni *post hoc* test.

Glucose tolerance test (2 g glucose/kg body weight, i.p.) in TNFR1^−/−^ mice or wild-type littermates performed 7 days after i.c.v. injection of vehicle or AβOs. Bar graph represents areas under the curves (AUC) in the time course plots (*n *=* *8 WT + Veh; 7 WT + AβOs; 7 TNFR^−/−^ + Veh; 8 TNFR^−/−^ + AβOs). Left panel: **P =* *0.0049, ***P <* *0.0001, two-way ANOVA followed by Bonferroni *post hoc* test; right panel: *P =* *0.0001, one-way ANOVA followed by Bonferroni *post hoc* test.

Western blot analysis of phospho-IKKβ (D; *n *=* *6 WT + Veh; 6 WT + AβOs; 4 TNFR^−/−^ + Veh; 6 TNFR^−/−^ + AβOs) and IRS-1pSer^636^ levels (E; *n *=* *6 WT + Veh; 5 WT + AβOs; 6 TNFR^−/−^ + Veh; 5 TNFR^−/−^ + AβOs) in the hypothalamus of wild-type (WT) or TNFR1^−/−^ mice 10 days after i.c.v. injection of vehicle or AβOs. Representative images from Western blot experiments were always run on the same gels but represent noncontiguous lanes. In (D), *P =* *0.0088, Student's *t*-test; in (E), *P =* *0.0428, one-way ANOVA followed by Bonferroni *post hoc* test.

Adult Swiss mice pre-treated with minocycline or PBS received a single i.c.v. injection of vehicle or 10 pmol AβOs, and hypothalamic levels of mRNA for AgRP (F; *n *=* *5 Veh; 6 AβOs; 5 Veh + Mino; 4 AβOs + Mino) and NPY (G; *n *=* *14 Veh; 13 AβOs; 8 Veh + Mino; 9 AβOs + Mino) were analyzed 7 days after injection. In (F), *P =* *0.0097, one-way ANOVA followed by Bonferroni *post hoc* test; in (G), *P =* *0.0219, one-way ANOVA followed by Bonferroni *post hoc* test.

Glucose tolerance test (GTT) in APP/PS1 mice before and after i.c.v. injection of infliximab (0.2 μg daily for 4 days). Bar graph represents areas under the curves (AUC) in the time course plots (*n *=* *9 animals/group). Left panel: *P =* *0.0177, two-way ANOVA followed by Bonferroni *post hoc* test; right panel: *P =* *0.0327, paired *t*-test. Data information: Data are expressed as means ± SEM. To assess statistical significance, AβO-injected mice were compared to vehicle-injected mice. Source data are available online for this figure.

We recently reported that aberrant TNF-α signaling in the hippocampus mediates impaired neuronal insulin signaling, synapse deterioration and memory loss in mice receiving i.c.v. infusions of AβOs (Bomfim *et al*, 2012; Lourenco *et al*, 2013). In addition, pioneering studies have established that activation of pro-inflammatory TNF-α is a key mechanism leading to peripheral insulin resistance in diabetes (Hotamisligil & Spiegelman, 1994; Hotamisligil *et al*, 1996; Gregor & Hotamisligil, 2011) and that inhibition of hypothalamic inflammation prevents peripheral insulin resistance (Milanski *et al*, 2012). Furthermore, our *in vitro* results indicated that TNF-α mediates AβO-induced eIF2α-P (Fig3E). Thus, we hypothesized that the TNF-α pathway might be involved in AβO-induced deregulation of glucose homeostasis in mice. To this end, we investigated the effects of i.c.v.-injected AβOs in TNF-α receptor 1 knockout mice (Romanatto *et al*, 2009). AβOs failed to induce glucose intolerance in TNFR1^−/−^ mice (Fig7C). In metabolic disorders, ER stress has been linked to insulin resistance and pro-inflammatory TNF-α signaling (Ozcan *et al*, 2006; Steinberg *et al*, 2006). TNF-α signaling has further been shown to activate intracellular stress kinases, including IKKβ (Hotamisligil *et al*, 1996; Cai *et al*, 2005). i.c.v.-injected AβOs triggered IKKβ activation and IRS-1pSer^636^ in the hypothalamus of wild-type mice, but failed to do so in TNFR1^−/−^ mice (Fig7D and E). Because whole-body TNF-α signaling would be expected to be affected in TNFR1^−/−^ mice, and to further investigate the specific role of brain TNF-α signaling in deregulation of glucose metabolism, we performed additional experiments in mice that were treated i.c.v. with infliximab. We found that AβOs failed to trigger glucose intolerance in mice that were previously treated with infliximab (Supplementary Fig S6).

Our recent studies on the effects of oligomers in the hippocampus indicate that, in addition to a direct effect on neurons, oligomers also seem to impact microglial cells, the cellular components of the innate immune system in the brain, to induce increased TNF-α levels and to deregulate hippocampal function (Lourenco *et al*, 2013). Therefore, we decided to test whether a similar indirect effect of oligomers might lead to alterations in AgRP and NPY expressions in the hypothalamus. To this end, we carried out experiments in mice that had been treated intraperitoneally with minocycline, an antibiotic known to prevent microglial activation and polarization to an M1 proinflammatory profile. For reasons that are unclear to us, minocycline treated-mice injected with vehicle showed increased hypothalamic expression of AgRP and NPY (albeit not statistically significant when compared to vehicle-injected mice) (Fig7F and G). Importantly, AβOs failed to induce increases in AgRP and NPY levels in mice that had been treated with minocycline (Fig7F and G). This indicates that oligomers act on microglial cells, which likely secret soluble factors (including TNF-α) to increase neuronal AgRP and NPY expressions. Results thus indicate that a crosstalk between neuronal and microglial cells is key to the effects of AβOs in the hypothalamus. Finally, we tested whether infliximab treatment would alleviate glucose intolerance in APP/PS1 mice. To this end, we performed i.c.v. injections of infliximab in APP/PS1 mice and found that infliximab rescued glucose intolerance in transgenic mice (Fig7H).

## Discussion

Diabetes and AD are chronic degenerative diseases increasing in prevalence in aging populations worldwide. Although clinical and epidemiological studies have linked AD to diabetes, with each disease increasing the risk of developing the other, the mechanisms of pathogenesis connecting them at the molecular and cellular levels remain to be elucidated. In particular, why AD patients present increased probability of developing diabetes is unknown. Here, we show that AβOs, toxins that accumulate in the AD brain and have been linked to neuronal dysfunction in brain areas related to learning and memory, impact the hypothalamus of mice and macaques. Intriguingly, infusion of AβOs in the brain triggers peripheral insulin resistance in mice. Alterations in peripheral glucose homeostasis were further detected in two transgenic mouse models of AD. These results provide initial evidence implicating Aβ oligomers in the biological mechanisms underlying the clinical observations linking AD to diabetes.

Numerous studies have investigated the impact of AβOs in memory centers, specially the hippocampus (Ferreira & Klein, 2011), known to be fundamentally involved in the acquisition, consolidation and recollection of new memories. This is because AD is classically recognized as a disease of memory, and indeed memory-related brain regions have long been known to be affected in the course of disease (Walsh *et al*, 2002; Chhatwal & Sperling, 2012). However, early studies indicated that other brain regions, not necessarily involved in learning and memory, might also be affected in AD. For example, *postmortem* analysis of AD brains identified Aβ deposits in the hypothalamus (Ogomori *et al*, 1989; Standaert *et al*, 1991), and evidence of peripheral glucose intolerance in AD patients has been reported (Craft *et al*, 1992). More recently, voxel-based morphometry analysis showed reduced hypothalamic volume and a decreased number of orexin neurons in AD patients compared to healthy controls (Loskutova *et al*, 2010; Fronczek *et al*, 2012). Furthermore, hyperglycemia and hyperinsulinemia were shown to positively correlate with the development of AD pathology (Matsuzaki *et al*, 2010). In transgenic mouse models of AD, obesity-induced insulin resistance is exacerbated (Takeda *et al*, 2010; Jimenez-Palomares *et al*, 2012). Collectively, these observations raise the intriguing possibility that the neuroendocrine axis, including the hypothalamus, may be affected in AD. However, studies investigating the mechanisms underlying such clinical and *postmortem* observations are lacking. Using different experimental models, including cell-based assays, mice and macaques that received i.c.v. injections of AβOs, we now report that the hypothalamus is affected by AβOs.

In both mice and macaques, i.c.v. infusion of AβOs induced hypothalamic inflammation and eIF2α-P, recently implicated as important pathogenic events in the onset of peripheral insulin resistance in metabolic disorders (Zhang *et al*, 2008; Denis *et al*, 2010; Arruda *et al*, 2011; Thaler *et al*, 2012). Interestingly, while in mice we found a transient increase in hypothalamic eIF2α-P levels following a single i.c.v. injection of oligomers, persistently elevated eIF2α-P levels were found in macaques after a series of AβO injections. This suggests that persistently elevated oligomer levels in the brain may induce prolonged effects in eIF2α-P. AβOs further induced IRS-1 inhibition (IRS-1pSer) in the hypothalamus of mice. It is noteworthy that oligomers failed to trigger both hypothalamic IKKβ activation and IRS-1pSer in TNFR1^−/−^ mice. Results thus indicate that AβO-induced TNF-α/pIKK deregulation is directly linked to disrupted insulin signaling in the hypothalamus.

Activated JNK and PKR were recently implicated in AβO-induced defective hippocampal insulin signaling (Bomfim *et al*, 2012; Lourenco *et al*, 2013). However, at the time points investigated (4 h or 7 days post-AβO injection), no differences in pJNK and pPKR levels were detected in the hypothalamus of AβO-injected mice compared to vehicle-injected mice. Further, no changes were detected in other markers of ER stress (4 h post-AβO injection), including phospho-PERK, IRE1a-pSer724, ATF6 and Grp78. We note that future studies aimed to analyze in more detail the time course of changes in levels of ER stress markers may provide additional insight into the mechanisms by which AβOs instigate hypothalamic deregulation.

Transient hypothalamic ER stress has been shown to induce increased plasma levels of noradrenaline in mice, and this was reported to be sufficient to induce peripheral glucose intolerance in mice (Purkayastha *et al*, 2011). Consistent with that interesting study, we found that prevention of brain ER stress by i.c.v. administration of TUDCA normalized plasma noradrenaline levels and blocked AβO-induced peripheral glucose intolerance. Moreover, AβOs failed to induce glucose intolerance, hypothalamic IKKβ activation and IRS-1 inhibition in TNFR1^−/−^ mice, or glucose intolerance in mice treated i.c.v. with infliximab. These results suggest that brain ER stress and inflammation underlie alterations in peripheral glucose homeostasis induced by AβOs, and indicate that AβOs hijack key signaling pathways in the CNS to deregulate peripheral glucose handling.

We recently demonstrated that i.c.v.-injected AβOs disrupt insulin signaling and induce inflammation in the hippocampus of mice and macaques (Bomfim *et al*, 2012; Ledo *et al*, 2013; Lourenco *et al*, 2013). AβO-induced abnormal hippocampal TNF-α signaling was found to be directly linked to synapse deterioration and cognitive impairment (Lourenco *et al*, 2013). It is thus possible that, in the AD brain, progressive accumulation of Aβ oligomers (due to elevated Aβ production or reduced clearance) brings about different functional outcomes in different brain regions. While the impact of AβOs in the hippocampus involves inflammation, ER stress and synapse deterioration, leading to memory deficits, AβO-induced inflammation and eIF2α-P in the hypothalamus may be especially relevant in terms of disrupting hypothalamic insulin signaling. The hypothalamus is well known for its ability to respond to changes in circulating insulin levels by regulating food ingestion (Sanchez-Lasheras *et al*, 2010). We found that an acute i.c.v. injection of insulin failed to suppress short-term food ingestion in AβO-injected mice, suggesting that AβOs rendered the hypothalamus resistant to insulin. Remarkably, activation of a hypothalamic inflammatory pathway similar to the pathway we report in our model has been implicated as a central mechanism regulating energy imbalance in obese mice, and its suppression has been proposed to represent a potential strategy to combat obesity-related diseases (Zhang *et al*, 2008). These findings further indicate that AβO- and obesity-induced hypothalamic inflammation share common pathogenic pathways.

Current findings indicate that AgRP and NPY levels remained unaltered in AβO-injected mice that had been treated with minocycline, suggesting that oligomers impact microglial cells, the cellular components of the innate immune system in the brain, likely inducing secretion of soluble factors (including TNF-α) to increase neuronal AgRP and NPY expressions. Furthermore, it is noteworthy that eIF2α-P does not depend on direct binding of oligomers to individual neurons, as elevated eIF2α-P levels were detected in neurons regardless of whether or not they had oligomers bound to their dendrites. Therefore, AβOs do not seem to act directly on neurons to induce phosphorylation of eIF2α. Rather, it is likely that a crosstalk between neurons and microglia leads to elevated levels of TNF-α, causing activation of neuronal TNF-α/eIF2α signaling to deregulate hypothalamic function. We note that similar observations were made in studies of the effects of AβOs on hippocampal cells (Lourenco *et al*, 2013).

We showed that no alterations in peripheral glucose homeostasis were detected 12 h after an i.c.v. injection of AβOs (Supplementary Fig S2C), but markers of hypothalamic inflammation were found to be elevated as soon as 4 h after AβO infusion. This supports the notion that hypothalamic inflammation precedes and may lead to peripheral metabolic alterations, a possibility that deserves further investigation. In this regard, an interesting recent study reported that, unlike inflammation in peripheral tissues, which develops as a consequence of obesity, hypothalamic inflammatory signaling is evident in rats within 1 to 3 days of feeding on a high-fat diet, prior to substantial weight gain (Thaler *et al*, 2012) and implicating hypothalamic inflammation in obesity pathogenesis (Thaler *et al*, 2013). We further note that AβO-induced deregulation of peripheral glucose homeostasis is similar in magnitude to the deregulation induced by a short period (7 days) of high-fat diet. Extending the findings of a recent study using APP/PS1 mice (Zhang *et al*, 2012), we found altered peripheral glucose homeostasis both in APP/PS1 mice and in 3xTg-AD mice, two different experimental models of AD.

Importantly, we further demonstrated that i.c.v. injections of infliximab rescued glucose tolerance in APP/PS1 mice, establishing that brain inflammation triggers alterations in peripheral glucose homeostasis in AβO-injected mice and in the APP/PS1 mouse model of AD. Intracerebroventricular infusion of infliximab in AD transgenic mice has been reported to reduce the number of amyloid plaques and phospho-tau levels (Shi *et al*, 2011a). Intrathecal administration of infliximab was further reported to improve cognition in one patient with AD (Shi *et al*, 2011b), and clinical trials are currently investigating the efficacy of infliximab in a wide range of pathologies, including major depression, obesity-associated insulin resistance and diabetic complications, among others (US National Institute of Health; http://clinicaltrials.gov/). However, infliximab does not cross the blood–brain barrier, and so far, it is important to note that anti-TNF-α strategies for AD require invasive forms of central administration, making this a difficult strategy to treat AD. Nevertheless, our results suggest that pharmacological or other approaches to prevent neuroendocrine dysfunction may provide novel therapeutics for metabolic deregulation in AD.

Our results demonstrate that brain accumulation of AβOs affects the hypothalamus and impacts peripheral metabolism by mechanisms similar to those underlying peripheral insulin resistance in type 2 diabetes and other metabolic diseases. Similar to what has been described in metabolic disorders (Rossmeisl *et al*, 2003; Thaler *et al*, 2013), i.c.v.-injected AβOs induce adipose tissue inflammation and impaired insulin-induced surface translocation of GLUT-4 in muscle cells. A previous study reported that a very high concentration of Aβ (10 μM) induced hepatic insulin resistance *in vitro* through a direct effect on hepatocytes (Zhang *et al*, 2012). However, in our experimental conditions, AβOs failed to cause alterations in peripheral glucose homeostasis when delivered via the caudal vein or by intraperitoneal injection in mice, ruling out a direct effect of AβOs on peripheral tissues. It is important to note that, besides the hypothalamus, other brain regions involved in neuroendocrine control might be also affected by AβOs. Whether AβOs indeed affect other brain regions responsible for the control of peripheral glucose homeostasis warrants further exploration.

In conclusion, our findings establish that i.c.v.-injected AβOs trigger inflammation in the hypothalamus and cause peripheral glucose intolerance and insulin resistance. Results support the emerging notion that pathological hypothalamic inflammation/ER stress leads to impaired peripheral glucose homeostasis. We propose that the impact of AβOs on the hypothalamus comprises a key novel pathological mechanism that disrupts metabolic homeostasis and leads to insulin resistance, revealing an important crosstalk between central and peripheral pathogenic mechanisms in AD. Our discovery that AβOs instigate hypothalamic deregulation draws attention to a brain structure that has been largely ignored to date in the study of AD pathogenesis, and highlights the importance of recognizing AD as a disease of both the brain and the periphery. As peripheral insulin resistance has been implicated in the development of AD (Janson *et al*, 2004; De Felice, 2013), current results suggest the existence of a vicious cycle, instigated by brain accumulation of AβOs, contributing to the development of both AD and metabolic disorders, including type 2 diabetes.

## Materials and Methods

### Preparation of Aβ oligomers

Oligomers were prepared from synthetic Aβ_1–42_ peptide (American Peptide, Sunnyvale, CA) as originally described Lambert *et al* (1998). The peptide was solubilized in hexafluoroisopropanol (HFIP) and the solvent was evaporated to produce dried films, which were subsequently dissolved in sterile anhydrous dimethylsulfoxide to make a 5 mM solution. This solution was diluted to 100 μM in ice-cold PBS and incubated overnight at 4°C. The preparation was centrifuged at 14,000 *g* for 10 min at 4°C to remove insoluble aggregates (protofibrils and fibrils), and the supernatants containing soluble Aβ oligomers were stored at 4°C. Protein concentration was determined using the BCA kit (Pierce, Deerfield, IL). Routine characterization of preparations was performed by size-exclusion chromatography and Western blotting using anti-Aβ 6E10 (Abcam, Cambridge, MA) or anti-Aβ oligomer NU1 (Lambert *et al*, 2007) monoclonal antibodies and, occasionally, by transmission electron microscopy, as previously described (Jurgensen *et al*, 2011; Sebollela *et al*, 2012; Figueiredo *et al*, 2013). Oligomers were used within 48 h of preparation.

### Mature hypothalamic neuronal cultures, immunocytochemistry, ROS and LDH release assays

Primary hypothalamic neuronal cultures were prepared from rat embryos (E16) according to the procedures established for hippocampal neuronal cultures (De Felice *et al*, 2007, 2009). Cultures were plated at a density of 70,000 cells/cm^2^ on poly-L-lysine-coated coverslips and were maintained in neurobasal medium with B27 supplement and L-glutamine (0.5 mM). After 14 days *in vitro,* cultures were incubated with vehicle or 500 nM AβOs for 3 h at 37°C. Infliximab was added 30 min prior to AβOs. For experiments designed to determine reactive oxygen species (ROS) formation, 20,000 cells/cm^2^ were plated directly on poly-L-lysine-coated wells of 96-well plates. After 18–21 days *in vitro,* cultures were incubated for 4 h at 37°C with vehicle or 500 nM AβOs. ROS formation was assessed using 2 μM of the fluorescent probe CM-H_2_DCFDA (Invitrogen, Carlsbad, CA), as previously described in De Felice *et al* (2007). CM-H_2_DCFDA is sensitive to the formation of various types of ROS, including peroxide, hydroxyl radical, peroxyl radicals and peroxynitrite. After 30 min of loading with the fluorescent probe, neurons were rinsed three times with warm PBS and two times with neurobasal medium without phenol red. Cells were immediately imaged on a Nikon Eclipse TE 300-U fluorescence microscope. At least three experiments with independent neuronal cultures were performed, each with triplicate well per experimental condition. Three images were acquired from randomly selected fields per well. Results obtained in independent experiments were combined to allow quantitative estimates of changes in neuronal ROS levels. Quantitative analysis of immunofluorescence data was carried using ImageJ (Windows version) using appropriate thresholding to eliminate background signal before histogram analysis, as described by De Felice *et al* (2007).

Immunocytochemistry was performed as previously described by De Felice *et al* (2009). Briefly, hypothalamic cultures were treated for 3 h at 37°C with 500 nM AβOs or equivalent volumes of vehicle and were fixed for 10 min with 4% paraformaldehyde containing 4% sucrose in PBS. Cells were blocked for 1 h with 10% normal goat serum in PBS and incubated at 4°C with monoclonal AβO-selective NU4 antibody (1:2,000; (Lambert *et al*, 2007)) overnight. Neurons were rinsed three times with PBS, permeabilized with 0.1% Triton X-100 for 5 min and incubated overnight at 4°C with anti-MAP2 (Santa Cruz Biotechnology, Santa Cruz, CA; 1:200, Cat#sc20172), anti-GFAP (DAKO, Carpinteria, CA; 1:200, Cat#Z-0334) or anti-phospho-eIF2α (Enzo Life Sciences, Farmingdale, NY; 1:200, Cat#BML-SA405) antibodies. After rinsing, neurons were incubated for 2 h at room temperature with Alexa Fluor-555 anti-mouse IgG and Alexa Fluor-488 anti-rabbit IgG (1:2,000). After washing, cells were mounted on coverslips using Prolong Gold Antifade with DAPI (Invitrogen) and were imaged on a Zeiss Axio Observer Z1 Microscope equipped with an Apotome module.

Measurement of lactate dehydrogenase (LDH) released to the medium was assessed as a cell death indicator. LDH was assayed by a commercial kit (Doles, Goiânia, Brazil) according to manufacturer's instructions. Briefly, culture medium was collected after exposure to AβOs (or vehicle) and LDH activity was measured. Absorbance was measured at 510 nm.

### Animals and intracerebroventricular (i.c.v.) injections

Male Swiss mice obtained from our own animal facility were 2.5–3 months old at the beginning of experiments. TNFR1^−/−^ female mice in a C57/BL6 background and wild-type littermates were obtained from the University of Campinas Breeding Centre (CEMIB). Six-month-old triple-transgenic (3xTg-AD) male mice and wild-type littermates were obtained from University of California Irvine (Xu *et al*, 2003). Nine- to thirteen-month-old APP/PS1 (seven males and two females) and littermate wild-type mice (six males and three females) were obtained from our own breeding facilities. Animals were housed in groups of five in each cage with free access to food and water, under a 12-h light/dark cycle, with controlled room temperature and humidity. Animals were randomly assigned to different experimental groups, and researchers conducting the experiments were blind to experimental condition. All procedures were performed in the light phase and followed the ‘Principles of Laboratory Animal Care’ (US National Institutes of Health) and were approved by the Institutional Animal Care and Use Committee of the Federal University of Rio de Janeiro (protocol IBqM 072-05/16) and UCI Institutional Animal Care and Use Committee. For i.c.v. injection of AβOs, animals were anesthetized for 7 min with 2.5% isoflurane (Cristália, São Paulo, Brazil) using a vaporizer system (Norwell, MA) and were gently restrained only during the injection procedure itself, as recently described in Figueiredo *et al* (2013). A 2.5-mm-long needle was unilaterally inserted 1 mm to the right of the midline point equidistant from each eye and 1 mm posterior to a line drawn through the anterior base of the eye (Laursen & Belknap, 1986; Figueiredo *et al*, 2011, 2013); see Supplementary Fig S1). Ten pmol of AβOs (concentration expressed in terms of Aβ monomers) or vehicle was injected in 30 s, in a total volume of 3 μl for Swiss mice. When C57/BL6 mice were used, 100 pmol of AβOs or vehicle was injected in 30 s in a total volume of 1 μl. Injection of 3 μl of a blue dye into the lateral ventricle of Swiss mice was performed to verify diffusion along the CSF circulation so as to reach the whole brain (Supplementary Fig S1). At the end of experiments, injection of blue dye in the same injection site used for AβOs or vehicle was employed to verify the accuracy of injection into the lateral ventricle. Mice showing any signs of misplaced injections or brain hemorrhage (∽5% of animals throughout our study) were excluded from further analysis.

In experiments using macaques, six female cynomolgus macaques (*Macaca fascicularis*; body weights 4.7–7.0 kg) were used. Macaques were maintained at the Centre for Neuroscience at Queen's University (Kingston, Canada) under the close supervision of a laboratory animal technician and the Institute veterinarian. All animals had a cannula implanted in the lateral ventricle by aseptic surgery. Anesthesia was induced by ketamine (10 mg/kg, intramuscular). During surgery, glycopyrrolate (0.013 mg/kg) and isoflurane (1–3%) were also used. Correct placement of the cannula was assessed by MRI. After a recovery period, three macaques received intracerebroventricular injections of 100 μg of AβOs (one injection per day every 3 days for 24 days). Three sham-operated macaques were used as controls. Upon completion of the experimental protocol, macaques were sedated with intramuscular ketamine (10 mg/kg) plus buprenorphine (0.01 mg/kg) for analgesia, followed by intravenous sodium pentobarbital (25 mg/kg), perfused with phosphate-buffered saline (PBS) followed by 4% paraformaldehyde in PBS; 4% paraformaldehyde in PBS containing 2.5% glycerol; PBS + 5% glycerol; and PBS + 10% glycerol. All procedures were approved by the Queen's University Animal Care Committee and were in full compliance with the Canada Council on Animal Care (Animal Care Protocol Original Munoz-2011-039-Or).

### Immunohistochemistry in macaque brain sections

Immunohistochemistry was performed using free-floating serial 40-μm-thick coronal sections in PBS containing 1% Triton X-100 incubated with 0.1 M citrate buffer, pH 6, at 60°C for 5 min. Endogenous peroxidase was inactivated by incubation of sections with 3% hydrogen peroxide in methanol for 2 h. Sections were then blocked with 5% bovine serum albumin (BSA) and 5% normal goat serum (NGS) in 1% Triton X-100 for 3 h at room temperature. Primary antibodies against phospho-eIF2α (Enzo Life Sciences; 1:200, Cat#BML-SA405), phospho-IKKβ (Abcam; 1:200, Cat#ab59195) and IκBα (Cell Signaling; 1:200, Cat#9242) were diluted in blocking solution, and sections were incubated at 4°C for 16 h, followed by incubation with biotinylated secondary antibody for 2 h at room temperature, and then processed using the Vectastain Elite ABC reagent (Vector Laboratories) according to manufacturer's instructions. The sections were washed in PBS and developed using DAB in chromogen solution, and counterstained with Harris' hematoxylin. Slides were mounted with Entellan (Merck) and imaged on a Zeiss Axio Observer Z1 microscope. Omission of primary antibody was routinely used to certify the absence of nonspecific labeling (data not shown). For immunofluorescence analysis, tissue autofluorescence was quenched by incubation with 0.06% potassium permanganate for 10 min at room temperature. Sections were blocked in 5% bovine serum albumin (BSA) and 5% normal goat serum (NGS) in 1% Triton X-100 for 3 h at room temperature. Primary antibody against AβOs (NU4; 1:300; (Lambert *et al*, 2007)) was diluted in blocking solution, and sections were incubated at 4°C for 16 h, followed by incubation with Alexa-555-conjugated anti-mouse secondary antibody (1:1,500) for 2 h at room temperature. Slides were mounted with Prolong Gold Antifade with DAPI (Invitrogen) and imaged on a Zeiss Axio Observer Z1 microscope equipped with an Apotome module to minimize out-of-focus light.

### Immunohistochemistry in mouse tissues

For GLUT-4 immunohistochemistry, mice received one i.c.v. injection of vehicle or 10 pmol AβOs. Seven days later, mice received one i.p. injection of either PBS or insulin (1 IU/kg body weight) and were killed by decapitation 15 min later. The *soleus* muscle was dissected and fixed in 4% paraformaldehyde. After 48 h, tissues were cryoprotected in sucrose (20-30%) and 20 μm sections were obtained in a cryostat (Leica CM1850). Sections were fixed with acetone for 30 min, washed twice with PBS and incubated for 1 h with rabbit polyclonal anti-GLUT-4 antibody (Abcam; 1:500, ab-654). Sections were then incubated with Alexa-555-conjugated anti-rabbit antibody (1:1,000; Invitrogen) for 1 h and mounted in Prolong Gold Antifade with DAPI (Invitrogen). Sections were imaged on a Zeiss Axio Observer Z1 microscope equipped with an Apotome module. Eight images were acquired per section, and integrated immunofluorescence intensity was determined using ImageJ software (Windows version). For adipose tissue immunohistochemistry, mice i.c.v. injected with vehicle or AβOs were killed 7 days after injection and samples of epididymal adipose tissue were removed and fixed in 4% paraformaldehyde. After 48 h, tissues were included into paraffin blocks, and 3 μm sections were obtained using a microtome and mounted in slides. For immunohistochemistry, slides were immersed in xylene for 10 min, sequentially rehydrated in absolute, 95 and 70% ethanol in water, and incubated with 3% H_2_O_2_ in methanol for inactivation of endogenous peroxidase. Antigens were reactivated by the treatment with 0.01 M citrate buffer for 40 min at 95°C. Slides were washed in PBS and incubated with CD68 antibody (Abcam; 1:200, Cat#ab125212) for 12–16 h at 2–8°C. After washing with PBS, slides were incubated with biotinylated secondary antibody for 1 h, washed twice with PBS and incubated with streptavidin-biotin peroxidase for 30 min. Slides were then covered with 3,3′ diaminobenzidine solution (0.06% DAB in PBS containing 2% DMSO and 0.018% H_2_O_2_) for 1 to 5 min or until a brown precipitate could be observed. Identical conditions and reaction times were used for slides from different animals to allow comparison between immunoreactivity densities. Reaction was stopped by immersion of slides in distilled water. Counterstaining was performed with Harris' hematoxylin. Four images were randomly acquired for each animal using a Zeiss Axio Observer Z1 microscope. An optical density threshold that best discriminated staining from background was obtained using NIH ImageJ 1.36b imaging software (NIH, Bethesda, MD).

Fluorojade (FJ) histochemistry was used as indicative of neuronal degeneration. The paraffin-embedded brain tissue sections were immersed into 100% ethanol for 3 min, then into 70% ethanol for 1 min and distilled water for 1 min. Slices were then immersed into 0.06% potassium permanganate solution for 10 min to suppress endogenous background signal, and slices were washed with distilled water for 1 min. Fluorojade B staining solution (10 ml of 0.01% Fluorojade B aqueous solution added to 90 ml of 0.1% acetic acid in distilled water) was added and slices were stained for 30 min. After staining, sections were rinsed three times with distilled water. Excess water was drained off, and the slides were cover-slipped with dibutylphthalate in xylene (D.P.X.) mounting media (Aldrich Chem. Co., Milwaukee, WI). Sections comprising the arcuate nucleus (Arc) and ventromedial hypothalamus (VMH) were examined on epifluorescence microscopes (Olympus Bx41 or Nikon Eclipse 50i). Positive staining controls consisted of sections from the hippocampus of a mouse i.c.v. injected with 36.8 nmol quinolinic acid and killed 24 h thereafter.

### Intraperitoneal glucose tolerance test (GTT)

Mice were fasted for 12 h and blood samples were collected from a tail incision. After collection of a baseline sample, mice received an i.p. injection of glucose (2 g/kg body weight). Blood glucose measurements were repeated at 15, 30, 45, 60 and 120 min after glucose injection, using a One-Touch® Ultra® Glucose Meter and strips (Johnson & Johnson). An additional measurement of blood glucose levels (180 min after glucose injection) was performed in experiments using 3xTg-AD mice. Mice with fasting glucose levels lower than 50 mg/dl or higher than 100 mg/dl, or whose plasma glucose levels did not increase at any time point after glucose injection were excluded from the study.

### Intraperitoneal insulin tolerance test (ITT)

Mice were fasted for 5 h and blood samples were collected from a tail incision. After collection of a baseline sample, mice received an i.p. injection of insulin (1 IU/kg body weight). Blood glucose measurements were repeated at 15, 30, 45 and 60 min after insulin injection, using a One-Touch® Ultra® Glucose Meter and strips (Johnson & Johnson). If blood glucose levels fell below 20 mg/dl, mice were immediately given an i.p. injection of glucose and were excluded from the experiment. Kitt was calculated as described by Ropelle *et al* (2010).

### Plasma insulin and leptin measurements

Mice were i.c.v.-injected with vehicle or AβOs and 7 days later were fasted for 3 h before being deeply anesthetized with 100 mg/kg ketamine and 10 mg/kg xylazine. After complete loss of reflex, blood samples were collected in EDTA-containing tubes and kept on ice until plasma separation (centrifugation at 3,000 *g* for 10 min at 4°C). Samples were kept at 4°C, and insulin detection and leptin detection were performed using the Ultra Sensitive Mouse Insulin ELISA kit and Mouse Leptin ELISA kit (both from Crystal Chem Inc, Downers Grove, IL).

### High-fat diet

Mice were maintained for 7 days on normal chow or a high-fat diet containing 55% of energy derived from fat, 29% from carbohydrates and 16% from protein, prepared as described (Romanatto *et al*, 2009; Ropelle *et al*, 2010).

### Treatment with tauroursodeoxycholic acid (TUDCA)

Mice received 5 μg TUDCA i.c.v. per injection. Injections were carried out 20 min prior to AβO injection, and at 2, 24 and 96 h thereafter. An extra TUDCA injection was given 12 h before the GTT, which was performed 7 days after AβO administration. Twenty-four hours after the GTT, mice were deeply anesthetized with 100 mg/kg ketamine and 10 mg/kg xylazine, and blood samples were collected by cardiac puncture in heparinized tubes. Plasma was separated by centrifugation at 3,000 *g* at 4°C for 10 min, and samples were used for noradrenaline quantification (as described below).

### Treatment with infliximab

Swiss mice were given a single i.c.v. injection of 2 μl of a 0.1 μg/μl solution of Infliximab 20 min prior to AβOs. In APP/PS1 mice, daily i.c.v. injections of 1 μl of a 0.2 μg/μl solution of Infliximab were administered for 4 days. The last injection was performed 12 h prior to the glucose tolerance test.

### Minocycline treatment

Swiss mice received daily i.p. injections of minocycline (25 mg/kg) for 3 days prior to i.c.v. injection of AβOs. Control mice received i.p. injections of PBS.

### Intracaudal injections

Animals were anesthetized with halothane and aseptically injected via the tail vein with 10 pmol AβOs or Dulbecco's PBS, in a final injection volume of 100 μl.

### Determination of accumulated food intake and intracerebroventricular insulin injection

Swiss mice were submitted to stereotaxic surgery for implantation of a cannula directed to the third ventricle, as described in Ropelle *et al* (2010). Mice were allowed to recover from surgery in their home cages for 4 days before being placed in individual metabolic cages. Animals then received i.c.v. injections of vehicle or AβOs, and food intake was measured every day at the same time for 7 days. Mice then received an i.c.v. injection of PBS or insulin (200 mU) at the beginning of the dark cycle, and food intake was determined by the difference between chow given to mice immediately after injection and the weight of remaining chow 12 h after.

### Noradrenaline extraction and quantification

Norepinephrine levels in plasma were measured by HPLC separation coupled with electrochemical detection (HPLC-ED). Perchloric acid was added to the plasma samples to a final concentration of 0.1 M. Samples were centrifuged (10,000 *g*) to remove precipitated proteins, and supernatants were used for automated injection into the HPLC. Fast isocratic separation was obtained using a reverse-phase LC-18 column (4.6 × 250 mm; Supelco) with the following mobile phase: 20 mM sodium dibasic phosphate, 20 mM citric acid, pH 2.64, containing 10% methanol, 0.12 mM Na_2_EDTA and 566 mg/l heptanesulfonic acid.

### Western blots

Four hours, 6 h or 7 days after i.c.v. injection of AβOs (as indicated in ‘Results’), mice were euthanized by decapitation and the hypothalamus and gastrocnemius muscle were rapidly dissected and frozen in liquid nitrogen. For total protein extraction, samples were thawed and homogenized in buffer containing 25 mM Tris–HCl, pH 7.5, 150 mM NaCl, 1% NP-40 (Invitrogen), 1% sodium deoxycholate, 0.1% SDS, 5 mM EDTA, 1% Triton X-100 and phosphatase and protease inhibitor cocktail (Pierce–Thermo Scientific, Rockford, IL). Protein concentration was determined using the BCA kit. Aliquots containing 30 μg protein were resolved by SDS–PAGE in 4–20% polyacrylamide gels (Invitrogen) and were electrotransferred to nitrocellulose or PVDF membranes for 1 h at 300 mA. Blots were blocked for 1 h with 5% non-fat dry milk in Tween-Tris buffer solution at room temperature or with Odyssey blocking buffer (Licor, Lincoln, NE; 1:2 dilution in Tween-Tris Buffer) and were incubated overnight at 2°C with primary antibodies diluted in blocking buffer. Molecular weight markers were run in one lane in every gel (Benchmark pre-stained protein ladder; Life Technologies). Primary antibodies used were IRS-1pSer^636^ (Santa Cruz; 1:200, Cat. #sc-33957), IRS-1pSer^312^ (Invitrogen; 1:200, Cat#44814-G and Cell Signaling; 1:1,000, Cat#2381), IRS-1pTyr^465^ (Santa Cruz; 1:200, Cat#sc-17194), total IRS-1 (Santa Cruz; 1:200. Cat#sc-559), pJNK (Thr^183^/Tyr^185^) monoclonal antibody (Cell Signaling; 1:1,000, Cat#9255S), JNK polyclonal antibody (Cell Signaling; 1:1,000, Cat#9252S), phospho-eIF2α (Enzo Life Sciences; 1:1,000, Cat#BML-SA405 and Cell Signaling; 1:1,000, Cat#9721), total eIF2α (Abcam; 1:1,000, Cat#ab5369 and Cell Signaling; 1:1,000, Cat#9722), pIKKβ (Abcam; 1:1,000; Cat#ab59195), total IKKβ (Abcam; 1:1,000, Cat#ab55404), IκBα (Cell Signaling; 1:1,000, Cat#9242), pNF-κB p65 (Ser^536^; Cell Signaling; 1:1,000, Cat#3031), total NF-κB p65 (Santa Cruz; 1:250, Cat#sc-372), p-PKR (Santa Cruz; 1:200, Cat#sc-101784), total PKR (Santa Cruz; 1:250, Cat#sc-366778), ATF6 (Abcam; 1:1,000, Cat#ab11909), GLUT-4 (Abcam; 1:500, Cat#ab654), PERKpThr981 (Santa Cruz; 1:500, Cat#sc-32577), total PERK (Abcam; 1:500, Cat#ab65142), GRP78 (Abcam; 1:500, Cat#ab53068), ATF4 (Sigma; 1:500, Cat#WH0000468M1), spliced and unspliced Xbp1 (Abcam; 1:500, Cat#ab37152), phospho-IRE-1 (Novus Biological; 1:1,000, Cat#NB100-2323), total IRE-1 (Novus Biological; 1:1,000, Cat#NB100-2324), β-tubulin III (Sigma-Aldrich, St. Louis, MO; 1:10,000, Cat#T8660) and β-actin (Cell Signaling; 1:10,000, Cat#12262). After overnight incubation with primary antibodies, membranes were incubated with horseradish peroxidase-conjugated secondary antibody (1:30–50,000), IRDye800CW- or IRDye680RD-conjugated secondary antibodies (Licor; 1:10,000) at room temperature for 2 h. Chemiluminescence was developed using SuperSignal West Femto (Thermo Fisher Scientific). Alternatively, fluorescence intensities were quantified in an Odyssey CLx apparatus (Licor).

### Nuclear-enriched fractions

For the preparation of nuclear extracts, hypothalamus of vehicle- or AβO-injected mice was homogenized in 0.1 ml hypotonic lysis buffer (10 mM Hepes, pH 7.9, 1.5 mM MgCl_2_, 10 mM KCl, 0.5 mM dithiothreitol plus a phosphatase and protease inhibitor cocktail) for 15 min at 4°C. Cells were then lysed by adding 0.5% Nonidet P-40. The homogenate was centrifuged (13,000 *g* for 5 min at 4°C), and supernatants containing the cytoplasmic extracts were stored at −80°C. The nuclear pellet was resuspended in 75 μl ice-cold hypertonic extraction buffer (20 mM Hepes, pH 7.9, 300 mM NaCl, 1.5 mM MgCl_2_, 0.25 mM EDTA, 25% glycerol, 0.5 mM dithiothreitol plus phosphatase and protease inhibitors). After 40 min of intermittent mixing, extracts were centrifuged (13,000 *g* for 20 min at 4°C), and supernatants containing nuclear proteins were saved. Total protein concentration was determined using the BCA kit. Aliquots containing 20 μg protein were resolved by SDS–PAGE in 4–20% polyacrylamide gels (Invitrogen) and were electrotransferred to nitrocellulose membranes for 1 h at 300 mA. Blots were processed and incubated with antibodies as described above.

### Whole-cell recording

Whole-cell patch-clamp recordings were performed in neurons of the Arc in brain slices of male Swiss mice (2–3 months old). During the recordings, neurons were maintained in hypothalamic slice preparations and data analyses were performed as previously described Frazao *et al* (2013). Mice were decapitated and the entire brain was removed. After removal, brains were immediately submerged in ice-cold, carbogen-saturated (95% O_2_ and 5% CO_2_) artificial cerebrospinal fluid (aCSF; 126 mM NaCl, 2.8 mM KCl, 1.2 mM MgCl_2_, 2.5 mM CaCl_2_, 1.25 mM NaH_2_PO_4_, 26 mM NaHCO_3_ and 5 mM glucose). Coronal sections from a hypothalamic block (250 μM thick) were cut on a Leica VT1000S vibratome and incubated in oxygenated aCSF at room temperature for at least 1 h before recording. Slices were transferred to the recording chamber and allowed to equilibrate for 10–20 min before recording. The slices were bathed in oxygenated aCSF (32–34°C) at a flow rate of ∽2 ml/min. The pipette solution for whole-cell recording was modified to include an intracellular dye (Alexa Fluor 488): 120 mM K-gluconate, 10 mM KCl, 10 mM HEPES, 5 mM EGTA, 1 mM CaCl_2_, 1 mM MgCl_2_, 2 mM (Mg)-ATP and 0.03 mM Alexa Fluor 488 hydrazide dye, pH 7.3. Infrared differential interference contrast was used to target and obtain the whole-cell recording of neurons at the Arc (Leica DM6000 FS equipped with a fixed stage and a Leica DFC360 FX high-speed monochrome digital camera). Electrophysiological signals were recorded using an Axopatch 700B amplifier (Molecular Devices), low-pass-filtered at 2–5 kHz and analyzed offline on a PC with pCLAMP programs (Molecular Devices). Recording electrodes had resistances of 2.5–5 MΩ when filled with the K-gluconate internal solution. Input resistance was assessed by measuring voltage deflection at the end of the response to a hyperpolarizing rectangular current pulse (500 ms of −10 to −50 pA). Membrane potential values were compensated to account for junction potential (−8 mV). Solutions containing insulin (50 nM) were typically perfused for 5 min as previously described by Hill *et al* (2010). The recorded cells were randomly chosen. Alexa Fluor 488 hydrazide dye was used to verify the position of the recorded cells related to the third ventricle. Only cells located laterally to the third ventricle at a maximal distance of up to 100 micrometers were recorded.

### RNA extraction and quantitative real-time PCR analysis

Hypothalamus and adipose tissue from vehicle- or AβO-injected mice were homogenized in 500 or 1,000 μl Trizol (Invitrogen), respectively, and RNA extraction was performed according to manufacturer's instructions. Purity and integrity of RNA were determined by the 260/280 nm absorbance ratio and by agarose gel electrophoresis. Only preparations with ratios >1.8 and no signs of RNA degradation were used. In adipose tissue samples, a 30-min-long incubation at 30°C was performed, the lipid layer was removed and discarded, and RNA extraction was performed in the water soluble phase. One μg RNA was used for cDNA synthesis using the SuperStrand III Reverse Transcriptase kit (Invitrogen). Expression of genes of interest was analyzed by qPCR on an Applied Biosystems 7500 RT–PCR system using the Power SYBR kit (Applied Biosystems). Glyceraldehyde-3-phosphate dehydrogenase (GAPDH) or actin was used as endogenous control. Primer pairs used are shown in Supplementary Table S1. Cycle threshold (*C*_*t*_) values were used to calculate fold changes in gene expression using the 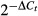 method. In all cases, reactions were performed in 15 μl reaction volumes.

### Statistical analysis

No previous statistical calculation was employed to determine sample size. Instead, sample size in our experiments was chosen based on usual procedures and best practices in the field. Gaussian distribution of data was assessed using the D'Agostino-Pearson normality test. Sample variances were assessed using the F test, when comparing two independent groups, and using Bartletts’ test and Brown-Forsythe test, when comparing three or more groups. Variances were equal between groups, except when stated otherwise. Glucose tolerance test curves were analyzed by two-way ANOVA followed by Bonferroni *post hoc* test. Two-tailed Student's *t*-test was performed when comparing two groups with comparable variances. All data in macaques shown unequal variances, and therefore, unpaired *t*-test with Welch's correction was applied. For experiments using APP/PS1 mice, a paired *t*-test was performed to compare groups before and after treatment with infliximab, since the same animals were assessed before and after drug administration. In Western blot experiments, a few lanes (indicated by a red ‘X’ symbol in the source data) were excluded from final analysis due to (i) excessive background, (ii) faint or undetectable bands in either phospho- or total proteins or (iii) fitting the mathematical definition of outliers.

## The paper explained

### Problem

Diabetes and Alzheimer's disease (AD) are chronic degenerative diseases increasing in prevalence in aging populations worldwide. Although clinical and epidemiological studies have linked AD to diabetes, with each disease increasing the risk of developing the other, why AD patients present increased probability of developing diabetes is unknown.

### Results

We demonstrate that AβOs, toxins that accumulate in the AD brain and have been linked to neuronal dysfunction in brain areas related to learning and memory, impact the hypothalamus of mice and macaques. The hypothalamus is a brain region that regulates glucose homeostasis in the body. Intriguingly, infusion of AβOs in the brain triggers glucose intolerance, insulin resistance and other manifestations of diabetes in mice. Similar alterations were observed in two transgenic mouse models of AD.

### Impact

Our discovery that AβOs instigate hypothalamic deregulation and glucose intolerance draws attention to a brain structure that has been largely ignored to date in the study of Alzheimer's pathogenesis. Since there is evidence that Alzheimer's patients present glucose intolerance, our results highlight the importance of recognizing Alzheimer's as a disease of both the brain and the periphery.

## Supporting Information

Supplementary Information

Review Process File
