# Supplementary material for: Alzheimer-associated Aβ oligomers impact the central nervous system to induce peripheral metabolic deregulation
Source: EMBO Mol Med. 2015 Jan 23;7(2):190–210. doi: 10.15252/emmm.201404183 (PMC4328648; doi:10.15252/emmm.201404183)
Supplement: Supplementary file 1 [file emmm0007-0190-sd1.doc]

**Clarke et al. - Supplementary Information**

Contents

| Supplementary Figure 1. Injection of blue dye into the lateral cerebral ventricle of mice…………………………………………………………………………………............ | 2 |
| --- | --- |
| Supplementary Figure 2. Effects of i.c.v.-injected AβOs or scrambled Aβ peptide *versus* short-term high-fat diet on peripheral glucose tolerance in mice…………………………... | 3 |
| Supplementary Figure 3. AβOs do not induce changes in hypothalamic levels of several ER stress markers and of phosphorylated JNK and PKR…………………........................... | 4 |
| Supplementary Figure 4. Cytoarchitecture of monkey hypothalamus……………............... | 5 |
| Supplementary Figure 5. AβOs do not affect electrophysiological properties of NPY-neurons……………………………………………………………………………………… | 6 |
| Supplementary Figure 6. I.c.v. treatment with infliximab prevents AβO-induced glucose intolerance in mice………………………………………………………………………….. | 7 |
| Supplementary Table 1. Primer sequences used for qPCR reactions………………………. | 8 |
| Supplementary References | 9 |
|  |  |


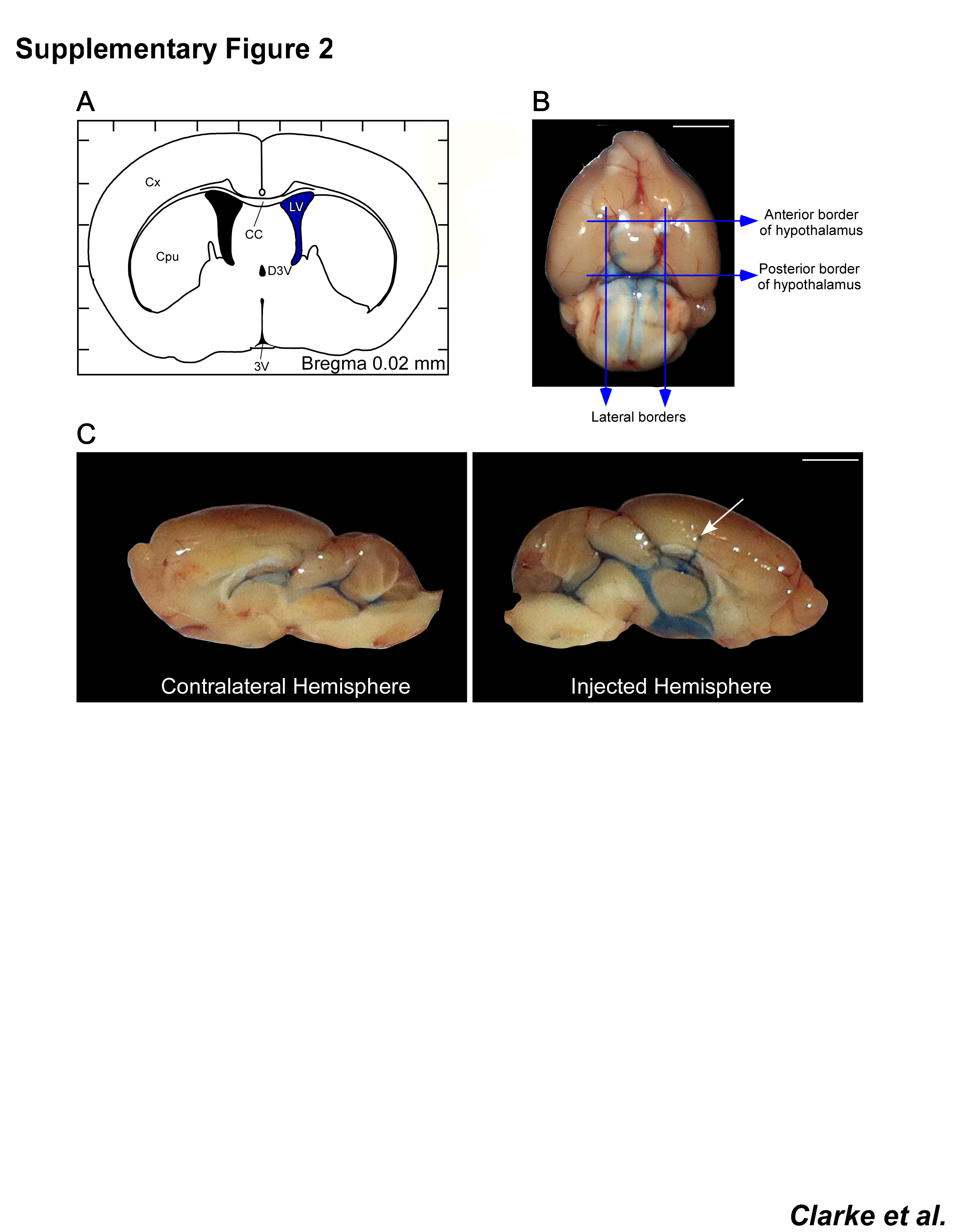


**Supplementary Figure 1. Injection of blue dye into the lateral cerebral ventricle of Swiss mice.** **A**, Scheme of a coronal section of the mouse brain (adapted from Paxinos and Franklin, 1997). Blue labeling represents the right lateral ventricle targeted by i.c.v. injection. Cx, cortex; CC, corpus callosum; Cpu, caudate putamen; LV, lateral ventricle; 3V, third ventricle; D3V, dorsal third ventricle. (**B, C**) bottom-up and sagital (along the midline) views, respectively, of a mouse brain after injection of 3 μl of blue dye into the lateral ventricle. Note the hypothalamus is completely surrounded by the dye (**B, C**). Arrow in **C** indicates the site of injection. Scale bar = 0.5 cm.


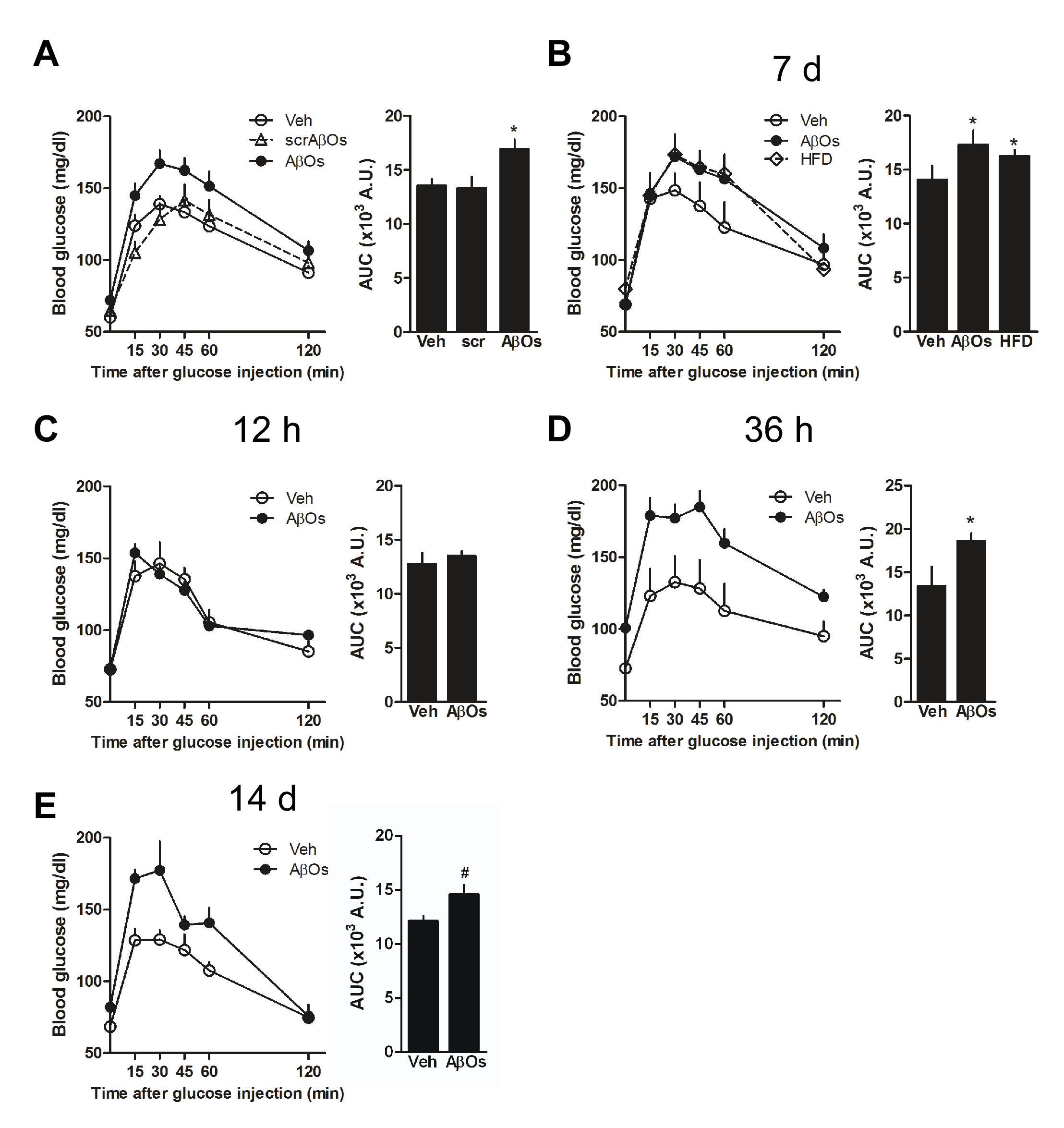


**Supplementary Figure 2.** **Effects of i.c.v.-injected AβOs or scrambled Aβ peptide *versus* short-term high-fat diet on peripheral glucose tolerance in mice. A,** Adult Swiss mice that received a single i.c.v. injection of vehicle, scrambled Aβ peptide (scrAβOs) or AβOs (10 pmol) were tested on a glucose tolerance test 7 days post-injection (2g glucose/kg body weight, i.p.; n = 13 Veh; 12 AβOs; 10 scrAβOs). **B,** Swiss mice fed a high-fat diet for 7 days were submitted to a glucose tolerance test (2g glucose/kg body weight, i.p.) and compared to Swiss mice that received a single i.c.v. injection of vehicle or AβOs (10 pmol; 7 days post-injection, n = 8 animals/group). **C**-**E,** Glucose tolerance tests (2 g glucose/kg body weight, i.p.) performed 12 h (**C**; n = 6 Veh; 9 AβOs), 36 h (**D**; n = 6 Veh; 9 AβOs) or 14 days (**E**; n = 6 animals/group) after i.c.v. injection of vehicle or AβOs (10 pmol). Bar graphs represent areas under the curves in the time-course plots of the glucose tolerance test. Data are expressed as means ± S.E.M. A, * p = 0.0155; B, * p = 0.0061 and 0.0037; D, * p = 0.0247; E, # p = 0.0665) one-way ANOVA followed by Bonferroni.


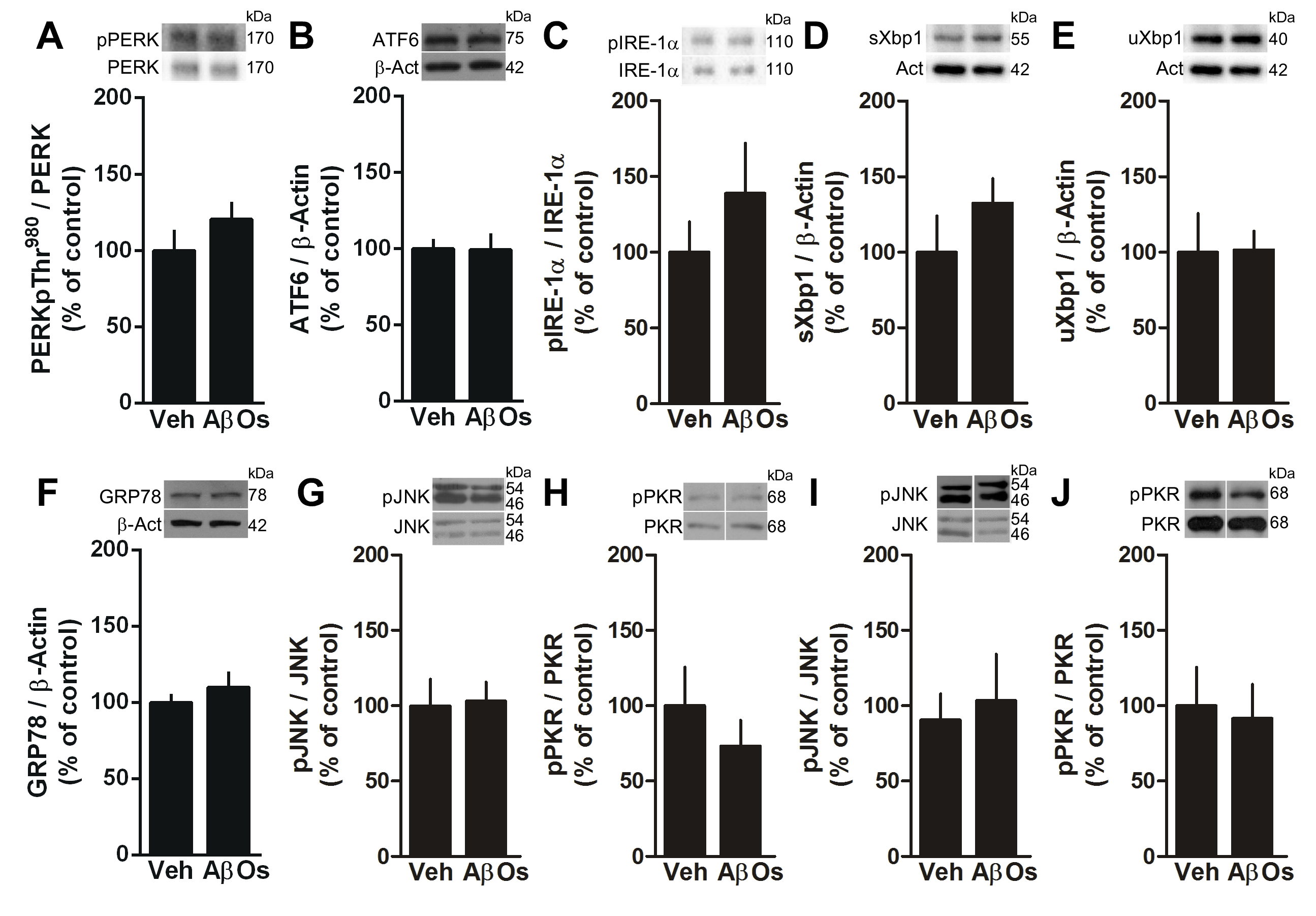


**Supplementary Figure 3. AβOs do not induce changes in hypothalamic levels of several ER stress markers and of phosphorylated JNK and PKR.** (**A**-**H**) Western blot analysis of hypothalamic levels of PERKpTHr980 (**A**; n = 5 Veh; 8 AβOs), ATF6 (**B**; n = 7 Veh; 8 AβOs), pIRE-1α (**C**; n = 7 animals/group), spliced (**D**; n = 5 Veh; 8 AβOs) and unspliced Xbp1 (**E**; n = 6 Veh; 8 AβOs), GRP78 (**F**; n = 7 Veh; 8 AβOs), pJNK (**G**; n = 4 Veh; 5 AβOs) and pPKR (**H**; n = 5 Veh; 4 AβOs) 4 h after a single i.c.v. injection of vehicle or 10 pmol AβOs in mice. (**I, J)**, Western blot analysis of hypothalamic levels of p-JNK (**I**; n = 5 animals/group) and pPKR (**J**; n = 4 Veh; 5 AβOs) 7 days after i.c.v. injection of vehicle or AβOs in mice. Graphs show densitometric data normalized by actin (**B,** **D-F**) or by the levels of total PERK (**A**), IRE-1 (**C**), JNK (**G** and **I**) or PKR (**H** and **J**). Data are expressed as mean ± S.E.M.

**
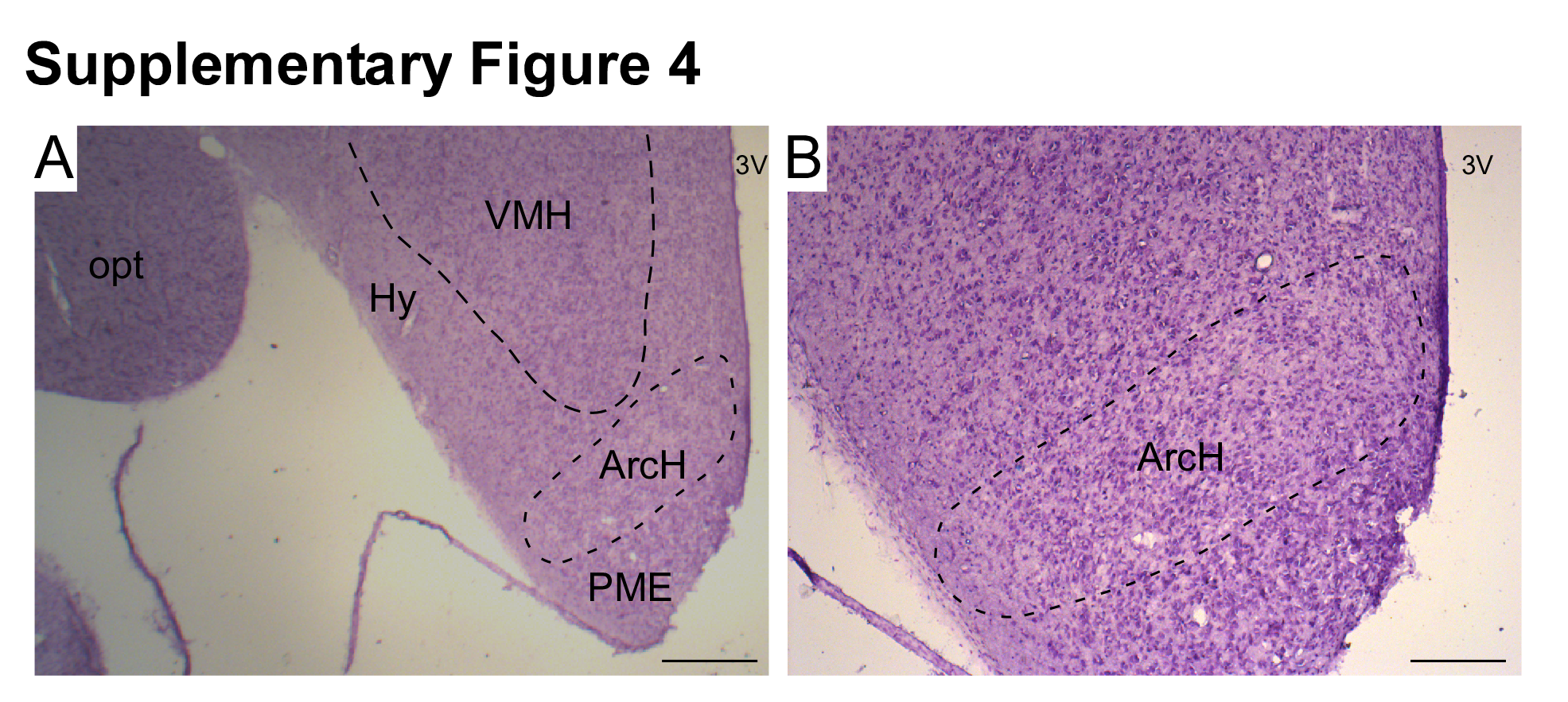
**

**Supplementary Figure 4. Cytoarchitecture of monkey hypothalamus.** Cresyl violet staining of a sham-operated monkey brain. 3V, third ventricle; VMH, ventromedial hypothalamus; PME, posterior medial eminence; opt, optic tract; Hy, hypothalamus; ArcH, arcuate nucleus of hypothalamus (Martin and Bowden, 1996). Scale bar = 200 μm in **A** and 100 μm in **B**.


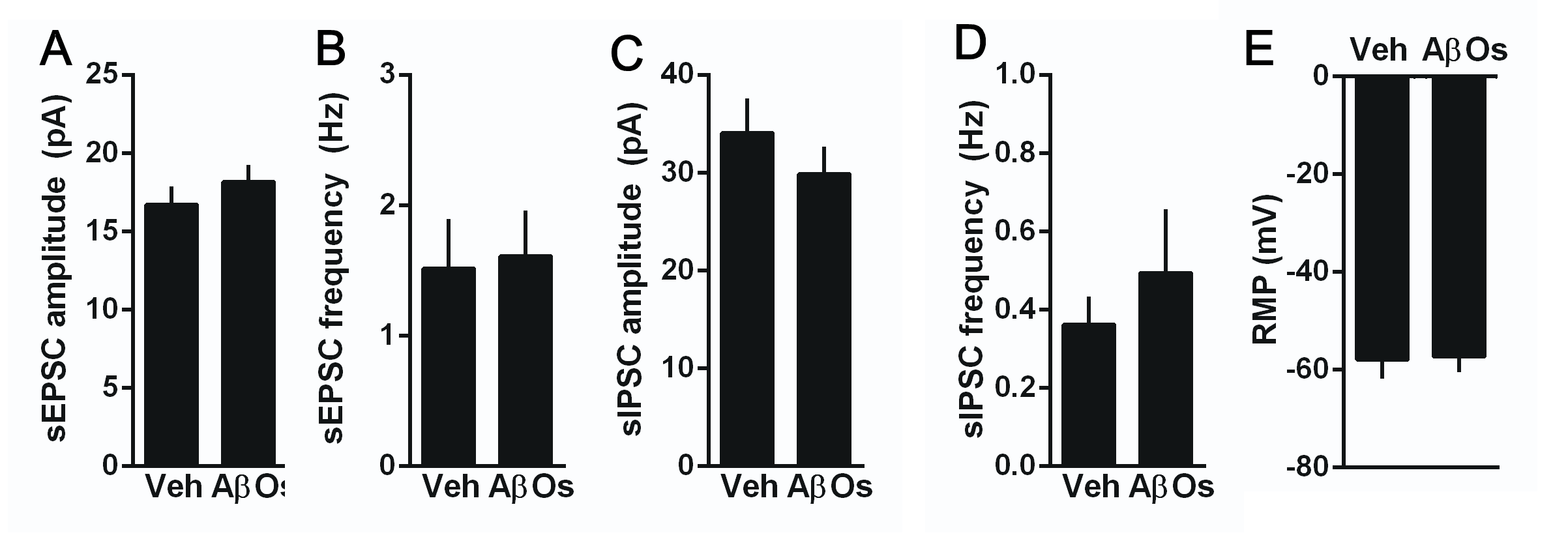


**Supplementary Figure 5. AβOs do not affect electrophysiological properties of NPY-neurons.** Swiss mice received a single i.c.v. injection of vehicle (Veh) or 10 pmol AβOs and their brains were used for whole-cell patch clamp recordings seven days after. (**A**-**E**) Coronal slices were used for recording of spontaneous activity of NPY neurons in the arcuate nucleus. No changes in amplitude or frequency of spontaneous excitatory (sEPSC) or inhibitory post-synaptic currents (sIPSC) were detected between groups. **E**, resting membrane potential of recorded cells. N = 20-21 slices obtained from 7 mice in each group, 3-4 cells recorded per slice. Data are expressed as mean ± S.E.M.


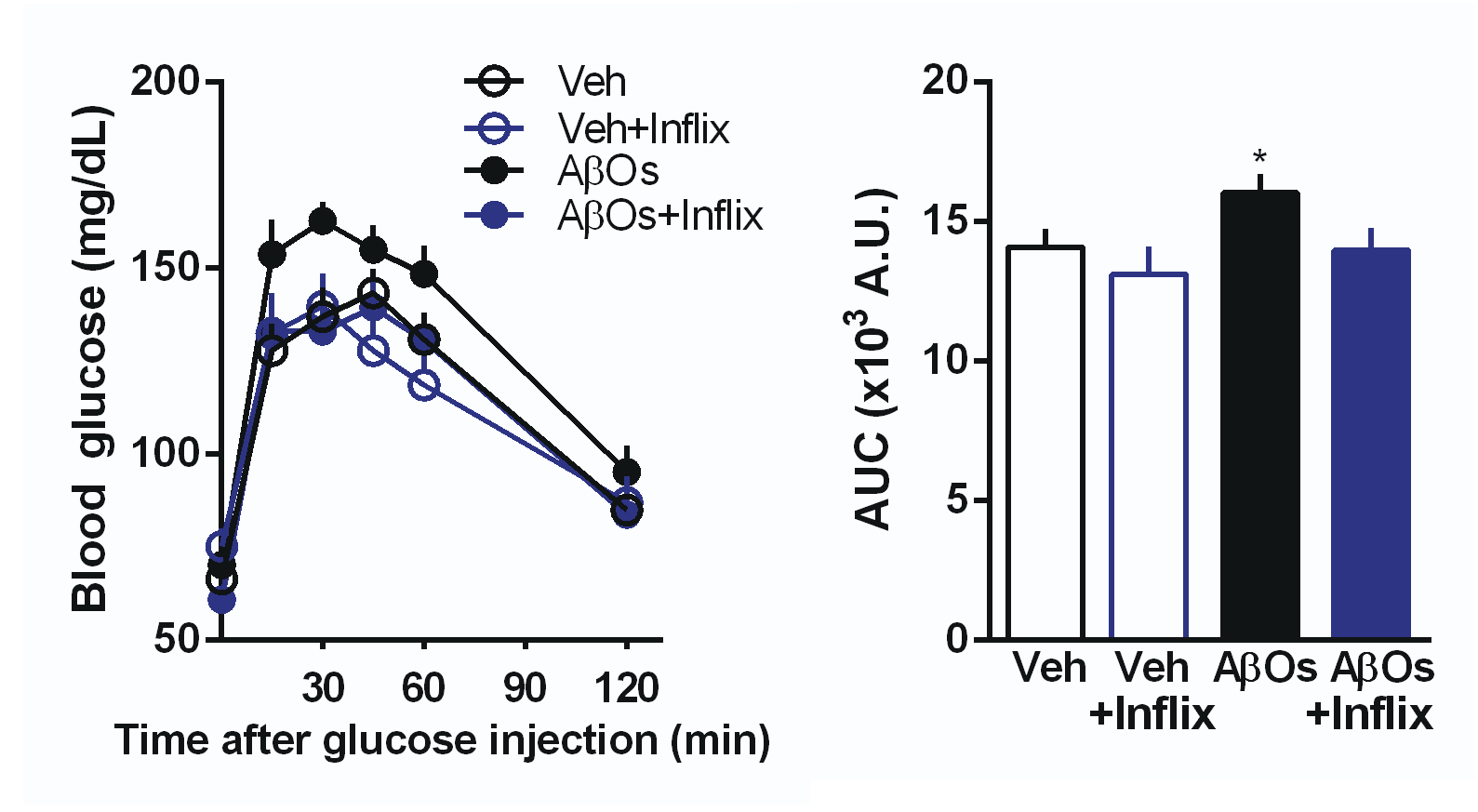


**Supplementary Figure 6. I.c.v. treatment with infliximab prevents AβO-induced glucose intolerance in mice.** Adult Swiss mice received a single i.c.v. injection of PBS or infliximab (0.2 μg) 20 min before receiving an i.c.v. injection of vehicle (Veh) or AβOs (10 pmol). Seven days post injection, mice were submitted to a glucose tolerance test (2g glucose/kg body weight, i.p.). Bar graphs represent areas under the curve in the time course plots of the glucose tolerance test. Data are expressed as means ± S.E.M. * p = 0.0494, Student’s *t*-test (Veh vs AβOs) (n = 12 Veh; 11 AβOs; 14 Veh+Inflix; 14 AβOs+Inflix).

**Supplementary Table 1. Primer sequences used for qPCR reactions.**

| Target gene | Forward primer | Reverse primer |
| --- | --- | --- |
| NPY | ATG CTA GGT AAC AAG CGA ATG G | TGT CGC AGA GCG GAG TAG TAT |
| POMC | ATG CCG AGA TTC TGC TAC AGT | TCC AGC GAG AGG TCG AGT TT |
| AgRP | ATG CTG ACT GCA ATG TTG CTG | CAG ACT TAG ACC TGG GAA CTC T |
| IL6 | TTC TTG GGA CTG ATG CTG GTG | CAG AAT TGC CAT TGC ACA ACT C |
| TNFα | CCC TCA CAC TCA GAT CAT CTT CT | GCT ACG ACG TGG GCT ACA G |
| Leptin | TGA GCT ATC TGC AGC ACG TT | TTC ACA CAC GCA GTC GGT AT |
| GLUT4 | AAA AGT GCC TGA AAC CAG AG | TCA CCT CCT GCT CTA AAA GG |
| Actin | GCC CTG AGG CTC TTT TCC AG | TGC CAC AGG ATT CCA TAC CC |
| GAPDH | AGG TCG GTG TGA TGA ACG GAT TTG | TGT AGA CCA TGT AGT TGA GGT CA |

**Supplementary References**

Martin RF, Bowden DM (1996) A stereotaxic template atlas of the macaque

brain for digital imaging and quantitative neuroanatomy. *NeuroImage* 4:

119 – 150

Paxinos G, Franklin K (1997) *The Mouse Brain in Stereotaxic Coordinates*. San

Diego, USA: Academic Press
