# Supplementary material for: Alzheimer-associated Aβ oligomers impact the central nervous system to induce peripheral metabolic deregulation
Source: EMBO Mol Med. 2015 Jan 23;7(2):190–210. doi: 10.15252/emmm.201404183 (PMC4328648; doi:10.15252/emmm.201404183)
Supplement: Supplementary file 2 [file emmm0007-0190-sd2.pdf]

EMBO Molecular Medicine

Alzheimer-associated Aβ oligomers impact the central nervous system to induce peripheral metabolic deregulation

Julia R. Clarke, Natalia M. Lyra e Silva, Claudia P. Figueiredo, Rudimar L. Frozza, Jose H. Ledo, Danielle Beckman, Carlos K. Katashima, Daniela Razolli, Bruno M. Carvalho, Renata Frazão, Marina A. Silveira, Felipe C. Ribeiro, Theresa R. Bomfim, Fernanda S. Neves, William L. Klein, Rodrigo Medeiros, Frank M. LaFerla, Jose B. Carvalheira, Mario J. Saad, Douglas P. Munoz, Licio A. Velloso, Sergio T. Ferreira, Fernanda G. De Felice.

Corresponding author: Fernanda De Felice, Federal University of Rio de Janeiro

Review timeline:

Submission date:	30 April 2014
Editorial Decision:	12 June 2014
Revision received:	08 November 2014
Editorial Decision:	05 December 2014
Revision received:	12 December 2014
Accepted:	17 December 2014

Transaction Report:

(Note: With the exception of the correction of typographical or spelling errors that could be a source of ambiguity, letters and reports are not edited. The original formatting of letters and referee reports may not be reflected in this compilation.)

Editor: Céline Carret

1st Editorial Decision 12 June 2014

Thank you for the submission of your manuscript to EMBO Molecular Medicine. We have now heard back from the two referees whom we asked to evaluate your manuscript.

Although the referees find the study to be of interest, they also raise a number of concerns mainly regarding the limited mechanistic insights that should be addressed in the next version of your manuscript. I will not get into experimental details, but we feel that the referees' reports are very clear and constructive and we would strongly encourage you to address all issues raised as recommended. We agree with referee 1 that a conditional KO would strengthen the manuscript, but we do not consider this as a precondition for the re-review of a resubmission.

Please note however that it is EMBO Molecular Medicine policy to allow only a single round of major revision and that, as acceptance or rejection of the manuscript will depend on another round of review, your responses should be as complete as possible.

EMBO Molecular Medicine has a "scooping protection" policy, whereby similar findings that are published by others during review or revision are not a criterion for rejection. Should you decide to submit a revised version, I do ask that you get in touch after three months if you have not completed it, to update us on the status.

Please also contact us as soon as possible if similar work is published elsewhere. If other work is

published we may not be able to extend the revision period beyond three months.

I look forward to receiving your revised manuscript.

***** Reviewer's comments *****

Referee #1 (Remarks):

The manuscript entitled "Alzheimer-associated A oligomers impact the central nervous system to induce peripheral metabolic deregulation" showed the evidence that central A oligomers can trigger peripheral glucose intolerance likely through affecting hypothalamic neurons. The authors further showed that A O is responsible for the induction of both ER stress and inflammation, providing the potential cellular mechanisms of the central A O-induced peripheral glucose intolerance.
This is a very interesting study that revealed a novel pathogenic action of A O in the central nervous system to affect peripheral glucose homeostasis. The authors used multiple in vivo and in vitro models to support their hypothesis. The manuscript is also very well written and presented. However, there are still some important questions left unanswered in the current manuscript. These concerns are needed to be addressed to further improve the manuscript.

Major Points:
1. One of the major concerns is to demonstrate how exactly A O affects neurons. Despite a lot of data showing A O increases ROS, ER stress and inflammation levels in hypothalamus, the authors still didn't show how this might impair the function of hypothalamic neurons. Does A O in these in vitro and in vivo experimental models cause neuronal death or glial cell death, which might explain the peripheral metabolism alterations? Or does A O affect hypothalamic neuron electrophysiology? Although the authors showed AgRP and NPY expression in the hypothalamus is significantly increased in A O group, it is still unclear if it's a direct effect or secondary effect through other neurons or glia.
2. The data showed that A O can bind to hypothalamic neurons in vitro. Can A O also bind to glial cells? How does A O binding change the molecular signaling in hypothalamic neurons? The authors need to provide more convincing evidence to link A O binding to increased ER stress and inflammation.
3. To prove that central inflammation caused by A O is one of the key factors for peripheral glucose intolerance, the authors used the TNFR-/- mouse model. However, the whole body knockout mouse would apparently affect inflammation in a lot of other organs. Therefore, conditional knockout mouse models or other alternative strategies are recommended to assess the role of inflammation specifically in the CNS.

Minor points:
1. Just using phospho-eIF2 to assess ER stress is not convincing. The authors need to show other typical ER stress markers such as phospho-PERK, phospho-IRE1 , uncleaved and cleaved XBP1, etc.
2. It is better to show serum levels of leptin and insulin in the study to provide a more comprehensive view of the state of glucose homeostasis impairment.
3. In Figure 2G, please show total IRS1 protein and normalize phospho-IRS1 to total IRS1.
4. In Figure 2H, is Glut4 expression level in muscle changed or only membrane distribution of Glut4?
5. In Figure 4B, please show total protein level of eIF2 and again normalize the phosphorylated protein to the total.

Referee #2 (Comments on Novelty/Model System):

The paper uses multiple model to establish that ABOs trigger an inflammatory cycle in the brain to cause peripheral insulin resistance and diabetes. I think this study is i the interest of general audience and provides valuable insights into an important but understudied area.

Referee #2 (Remarks):

This is an interesting study showing that centrally administered A-beta oligomers induced peripheral insulin resistance through engaging inflammatory signaling in the central nervous system. The authors show that central administration of ABO oligomers activated IKK-NF-kB pathway and ER stress and blocking TNF signaling or the use of a chemical chaperone can prevent glucose intolerance. These are interesting studies and provide important insights into the link between Alzheimer's disease and diabetes by pointing to a common inflammatory etiology. The experiments are of high quality and the paper is well written.

I have few relatively minor suggestions for the authors to consider.

Can the glucose intolerance in the transgenic model of AD be also prevented by TNF blockade (i.e., antibody administration) or by TUDCA treatment?

Can the authors examine additional indicators of ER stress upon administration of ABO oligomers, in addition to eIF2a phosphorylation (such as PERK phosphorylation, XBP splicing etc) and consider examining some of these markers in TNFR1-/- mice. These could strengthen the conclusions.

Please cite appropriate references for JNK activation in obesity and insulin resistance and the demonstration of TUDCA as an agent that alleviates insulin resistance in obese models.

1st Revision - authors' response 08 November 2014

Referee #1 (Remarks):

“The manuscript entitled "Alzheimer-associated Ab oligomers impact the central nervous system to induce peripheral metabolic deregulation" showed the evidence that central AB oligomers can trigger peripheral glucose intolerance likely through affecting hypothalamic neurons. The authors further showed that AbO is responsible for the induction of both ER stress and inflammation, providing the potential cellular mechanisms of the central AbO-induced peripheral glucose intolerance.

This is a very interesting study that revealed a novel pathogenic action of AβO in the central nervous system to affect peripheral glucose homeostasis. The authors used multiple in vivo and in vitro models to support their hypothesis. The manuscript is also very well written and presented. However, there are still some important questions left unanswered in the current manuscript. These concerns are needed to be addressed to further improve the manuscript.

Major Points:

1. One of the major concerns is to demonstrate how exactly AbO affects neurons. Despite a lot of data showing AbO increases ROS, ER stress and inflammation levels in hypothalamus, the authors still didn't show how this might impair the function of hypothalamic neurons.”

--- We agree with the reviewer that it would be important to further delineate the mechanistic underpinnings of AbO-induced dysfunction in hypothalamic neurons. To better understand how AbOs impair the function of hypothalamic neurons, we have carried out additional *in vitro* and *in vivo* experiments (please see our responses to specific questions below).

"Does AbO in these in vitro and in vivo experimental models cause neuronal death or glial cell death, which might explain the peripheral metabolism alterations?"

--- This is a pertinent question, as cell death has been described to take place at later stages of AD pathology and it would be interesting to ask if our model of AβO injection might lead to death of hypothalamic cells. To test this hypothesis, we initially used primary hypothalamic cultures. In cultures exposed to AbOs for 3 hours, we found no evidence of cell death using the LDH assay. We have included these new results in new Fig 3D. We next carried out Fluorojade staining in brain tissue from vehicle- or AbO-injected mice (7 days after AbO injection). Results showed no evidence of cell degeneration in AβO-injected mice compared to vehicle-injected animals (new Fig. 6F). We appreciate the opportunity to include these new results indicating that, under our experimental conditions, AbOs do not induce death of hypothalamic neurons.

"Or does AbO affect hypothalamic neuron electrophysiology?"

--- To address this question, we have carried out electrophysiology experiments in mice injected with AbOs. We have performed whole-cell patch-clamp recordings in brain slices from AβO-injected. We targeted cells from the arcuate nucleus, a region enriched in NPY-neurons. No changes were detected in frequency or amplitude of either excitatory or inhibitory post-synaptic currents, or in resting membrane potential of the recorded neurons (Supplementary Fig. 5), suggesting that the mechanism by which AbOs induce functional deregulation of hypothalamic neurons does not include major alterations in their electrophysiological properties.

"Although the authors showed AgRP and NPY expression in the hypothalamus is significantly increased in AbO group, it is still unclear if it's a direct effect or secondary effect through other neurons or glia."

--- This is an interesting point raised by the reviewer. Our recent studies on the effects of oligomers in the hippocampus indicate that, in addition to a direct effect on neurons, oligomers also impact microglial cells, the cellular components of the innate immune system in the brain, to deregulate hippocampal function (Lourenco et al. 2013, Cell Metab.; Ledo et al., unpublished results; please see our response to point # 3 below). Therefore, we decided to test if a similar indirect effect of oligomers might lead to alterations in AgRP and NPY expression in the hypothalamus. To this end, we carried out experiments in mice that had been treated intraperitoneally with minocycline, an antibiotic known to prevent microglial activation and polarization to an M1 proinflammatory profile. For reasons that are unclear to us, minocycline treated-mice injected with vehicle showed increased hypothalamic expression of AgRP and NPY (albeit not statistically significant when compared to vehicle-injected mice) (Fig. 7F, G). Importantly, AbOs failed to induce increases in AgRP and NPY levels in mice that had been treated with minocycline (Fig. 7F, G). This indicates that oligomers act on microglial cells, which likely secret soluble factors (including TNF-a) to increase neuronal AgRP and NPY expression. Results thus indicate that a crosstalk between neuronal and microglial cells are key to the effects of AbOs in the hypothalamus.

"2. The data showed that AbO can bind to hypothalamic neurons in vitro. Can AbO also bind to glial cells?"

--- We note that even though our hypothalamic cultures are mostly composed of neurons (~60%), they also contain significant proportions of glial cells. To examine the possibility that AbOs could also bind to astrocytes, we double-labelled cultures with anti-GFAP and NU4 (an oligomer-sensitive monoclonal antibody). Results indicate that oligomers do not bind to astrocytes in hypothalamic cultures (New panel B on Figure 3), similar to previous findings with hippocampal neuronal cultures. (e.g., Lacor et al., 2007, J. Neurosci).

"How does AbO binding change the molecular signalling in hypothalamic neurons? The authors need to provide more convincing evidence to link AbO binding to increased ER stress and inflammation."

--- This is a very pertinent comment dealing with a central aspect of our study, and we appreciate the opportunity to elaborate on the mechanism involved in oligomer-induced eIF2a activation and

inflammation. Indeed, following the reviewer's comment, we realized that presentation of our findings in the original manuscript was somewhat misleading with respect to this particular point.

Data presented in the revised manuscript establish that eIF2a-P and inflammation are not necessarily dependent on direct binding of Ab oligomers to individual neurons, but rather take place via secreted TNF-α signalling. Results supporting this conclusion are:

1) In neuronal hypothalamic cultures exposed to AbOs, new results indicate that neurons exhibited elevated phospho-eIF2a levels (new panel E on Figure 3) regardless of whether or not they had oligomers bound to dendrites (new panel F on figure 3). It is well known that oligomers target dendritic spines of a specific subset of neurons in culture, rather than all neurons (Lacor et al., 2007, J Neurosci). Thus, our findings indicate that elevation of phospho-eIF2a does not depend on direct binding of oligomers to individual neurons, but rather is likely instigated by soluble factors released to the medium upon exposure of cultures to AbOs. We feel this is an important piece of information that should be explicitly presented in the manuscript, and highlighted this point in both Results and Discussion (page 8, lines 17-21; page 15, lines 25-29).

2) Using neuronal hypothalamic cultures, we found that infliximab prevented eIF2a phosphorylation (new Fig. 3E), without blocking oligomer binding to neurons (new panel G on Fig. 3). This observation indicates that, even when oligomers remain bound to neurons, phosphorylation of eIF2a can be prevented by blockade of TNF-a signalling. This additional piece of information is presented in new Fig 3 (page 8, lines 27-30).

3) As mentioned above, even though our hippocampal cultures are mostly composed of neurons, they also contain significant proportions of glial cells, which we believe are the main sources of TNF-a. A recent study from our group showed that microglial activation and TNF-a levels are increased in the brains of mice that received intracerebroventricular infusions of oligomers (Ledo et al., Mol. Psychiatry, 2013). Studies currently under way in our lab further indicate that purified microglial cultures robustly respond to Ab oligomers to produce TNF-a (Ledo et al., manuscript in preparation). These data suggest that, following exposure to oligomers, a crosstalk between microglia and neurons leads to elevated levels of TNF-a, causing activation of TNF-a/eIF2a signalling. This point is now highlighted in "Results" (page 15, lines 28-29).

4) As mentioned above, results presented in the revised version of the manuscript show that AgRP and NPY levels remained unaltered in AbO-injected mice that had been treated with minocycline, in contrast with control mice (New Fig. 7 F and G). This demonstrates that oligomers act on microglial cells, which likely secret soluble factors (including TNFa) to increase neuronal AgRP and NPY expression. Results thus indicate that a crosstalk between neuronal and microglial cells are key to the effects of AbOs in the hypothalamus.

Collectively, our results indicate that neuronal eIF2a phosphorylation is triggered by TNF-a pro-inflammatory signalling instigated by AbOs, rather than being a direct consequence of oligomer binding to individual neurons.

"3. To prove that central inflammation caused by AbO is one of the key factors for peripheral glucose intolerance, the authors used the TNFR-/- mouse model. However, the whole body knockout mouse would apparently affect inflammation in a lot of other organs. Therefore, conditional knockout mouse models or other alternative strategies are recommended to assess the role of inflammation specifically in the CNS."

--- We agree with the reviewer that a conditional TNFR KO would strengthen our conclusions. However, unfortunately we were unable to obtain such mice to perform this interesting experiment in the time frame for revision of this manuscript. As an alternative strategy to address this issue, we investigated the effect of AbOs in mice that had previously received an i.c.v. injection of infliximab, a TNF-a neutralizing monoclonal antibody. Supporting our findings with $TNFR^{-/-}$ mice, we found that AbOs failed to trigger glucose intolerance in infliximab-treated mice (Supplementary Fig. 6). Furthermore, as suggested by reviewer #2 (see below), we carried out experiments aimed to test whether glucose intolerance in a transgenic mouse model of AD could be attenuated by TNF-a blockade. Results showed that i.c.v. injections of infliximab rescued glucose tolerance in APP/PS1

mice (new Fig. 7H). Results establish that brain inflammation triggers alterations in peripheral glucose homeostasis in AbO-injected mice as well as in the APP/PS1 mouse model of AD.

"Minor points:

1. Just using phospho-eIF2a; to assess ER stress is not convincing. The authors need to show other typical ER stress markers such as phospho-PERK, phospho-IRE1, uncleaved and cleaved XBP1, etc."

--- Following the reviewer's recommendation, we have now extended our analysis to investigate levels of other components of the UPR 4 h after i.c.v. injection of AbOs (a time-point when we detected elevated phospho-eIF2a levels). Consistent with increased eIF2a-P, levels of ATF4, a downstream effector of eIF2α were increased in the hypothalamus of mice 4 h after i.c.v. injection of AbOs (new panel C on Fig. 4). Other ER stress markers analysed remained unaltered, including PERKpThr980, ATF6, IRE1α-pSer724, spliced Xbp1,and Grp78 (Supplementary Fig. 3A-F). We acknowledge that we have examined ER stress markers at a single time point (4 hours post AbO injection) and note that a more detailed investigation of the time course of changes in levels of ER stress markers in the hypothalamus might be informative (we have included a sentence to this effect in Results; page 9, lines 8-12). We feel, however, that this would be beyond the scope of the current study, and hope to present such a detailed additional investigation in a follow up study. Based on these new findings, we have revised the manuscript to explicitly state that we have detected elevations in eIF2a-P rather than using the broader term "ER stress" to describe our findings.

"2. It is better to show serum levels of leptin and insulin in the study to provide a more comprehensive view of the state of glucose homeostasis impairment."

--- We have now included measurements of serum levels of leptin and insulin in mice 7 days after i.c.v. injection of AbOs. We found no changes in serum levels of leptin and insulin under these conditions. We note that results presented in the original version of our manuscript indicate that the impairment in glucose tolerance induced by i.c.v. administration of AβOs was comparable to that verified in mice submitted to a high-fat diet (HFD) for 7 days (Supplementary Fig. 2B). In harmony with our results, previous studies have shown that plasma leptin and insulin levels are not affected in mice (wild type or ob/ob) submitted to a short-term (4-7 days) HFD, whereas glucose tolerance and insulin sensitivity are clearly impaired under the same conditions (e.g., El-Haschimi et al., JCI 2000; Ji et al., JBC 2014; Beyec et al., Int J Obesity, 2014). Further, short-term HFD induces increased epididymal WAT weight, adipocyte hypertrophy and increased transcript levels of TNF-a and IL-6 (e.g. Ji et al., JBC 2014; Lee et al., Diabetes, 2011), similar to our observations in mice injected i.c.v. with AbOs. We agree with the reviewer that including these observations would provide a more comprehensive view of the state of glucose homeostasis impairment in AbO-injected mice, and we have included these new results in the revised manuscript (new Fig. 2K and L).

"3. In Figure 2G, please show total IRS1 protein and normalize phospho-IRS1 to total IRS1."

--- Total IRS1 is now shown in new Fig. 2G and is used for data normalization.

"4. In Figure 2H, is Glut4 expression level in muscle changed or only membrane distribution of Glut4?"

--- We have now analysed Glut4 expression, as well as Glut 4 levels in muscle and found that Glut4 expression and protein levels remained unaltered (Fig. 2I,J).

"5. In Figure 4B, please show total protein level of eIF2a; and again normalize the phosphorylated protein to the total."

--- Total eIF2a is now shown in new Fig. 4B and used for data normalization.

© EMBO

Referee #2 (Comments on Novelty/Model System):

"The paper uses multiple model to establish that ABOs trigger an inflammatory cycle in the brain to cause peripheral insulin resistance and diabetes. I think this study is in the interest of general audience and provides valuable insights into an important but understudied area."

Referee #2 (Remarks):

"This is an interesting study showing that centrally administered A-beta oligomers induced peripheral insulin resistance through engaging inflammatory signalling in the central nervous system. The authors show that central administration of ABO oligomers activated IKK-NF-kB pathway and ER stress and blocking TNF signalling or the use of a chemical chaperone can prevent glucose intolerance. These are interesting studies and provide important insights into the link between Alzheimer's disease and diabetes by pointing to a common inflammatory etiology. The experiments are of high quality and the paper is well written.

I have few relatively minor suggestions for the authors to consider.

1) Can the glucose intolerance in the transgenic model of AD be also prevented by TNF blockade (i.e., antibody administration) or by TUDCA treatment?"

--- We thank the reviewer for this very nice suggestion. Because our collaborators from UC Irvine in whose lab the experiments with 3XTg mice included in the original manuscript had been carried out were not able to provide the mice needed for this additional experiment, we have performed the suggested experiment using another transgenic mouse model of AD (APP/PS1 mice). We first verified that this different strain of mice also presents glucose intolerance (new Fig. 1E). Remarkably, glucose intolerance in APP/PS1 mice was rescued by i.c.v. treatment with infliximab, a TNF-a neutralizing antibody (new Fig. 7H). We further exploited an alternative strategy to address the role of central inflammation in triggering peripheral glucose intolerance. For that sake, we tested the ability of infliximab to block peripheral glucose intolerance induced by i.c.v. AbOs. Significantly, AbOs failed to trigger glucose intolerance in mice that previously received an i.c.v. injection of infliximab (Supplementary Fig. 6). Results establish that brain inflammation triggers deregulation of peripheral glucose homeostasis in AbO-injected mice as well as in the APP/PS1 mouse model of AD.

"2) Can the authors examine additional indicators of ER stress upon administration of ABO oligomers, in addition to eIF2a phosphorylation (such as PERK phosphorylation, XBP splicing etc) and consider examining some of these markers in TNFR1-/- mice. These could strengthen the conclusions."

--- This is an interesting suggestion also given by reviewer #1. We have now extended our analysis to investigate levels of other components of the UPR 4 h after i.c.v. injection of AbOs (a time-point when we detected elevated phospho-eIF2a levels). Consistent with increased eIF2a-P, levels of ATF4, a downstream effector of eIF2α were increased in the hypothalamus of mice 4 h after i.c.v. injection of AbOs (new panel C on Fig. 4). We also found a small trend of increase (albeit not statistically significant) in hypothalamic levels of XBP1. Other ER stress markers analysed remained unaltered, including IRE1a-pSer724, ATF6 and Grp78 (new data presented in Supplementary Fig. 3A-F). We acknowledge that we have examined ER stress markers at a single time point (4 hours post AbO injection) and note that a more detailed investigation of the time course of changes in levels of ER stress markers in the hypothalamus might be informative (we have included a sentence to this effect in Results; page 9, lines 8-12). We feel, however, that this would be beyond the scope of the current study, and hope to present such a detailed additional investigation in a follow up study.

Regarding the suggestion to examine ER stress markers in TNFR1$^{-/-}$ mice, we strived to perform this experiment but had problems with our supply of such mice. Thus, we were unable to accomplish

this in time for revision of the current manuscript. We feel this is an excellent point and plan on further investigating this in future experiments.

"3) Please cite appropriate references for JNK activation in obesity and insulin resistance and the demonstration of TUDCA as an agent that alleviates insulin resistance in obese models."

--- We apologize for the omission. We have now cited the appropriate articles.

2nd Editorial Decision 05 December 2014

Thank you for the submission of your revised manuscript to EMBO Molecular Medicine. We have now received the enclosed report from the referee who was asked to re-assess it. As you will see the reviewer is now supportive and I am pleased to inform you that we will be able to accept your manuscript pending final editorial amendments.

Please submit your revised manuscript within two weeks.

I look forward to reading a new revised version of your manuscript as soon as possible.

***** Reviewer's comments *****

Referee #1 (Remarks):

This is the revision of the manuscript entitled "Alzheimer-associated A oligomers impact the central nervous system to induce peripheral metabolic deregulation".

In the revision, the authors addressed all of the points this reviewer raised concerning the experimental design and interpretation of the data. With additional data, the authors further addressed that 1) A O does not directly affect electrophysiology and survival of hypothalamic neurons; 2) A O is likely acting through microglia, which secrets soluble factors including TNF-alpha to indirectly affect neuronal functions in hypothalamus; 3) regional blockade of TNF-alpha signaling via i.c.v. injection of infliximab indeed reverse the A O-induced glucose intolerance.

These additional studies lead to further and deeper mechanistic understanding of central A O-induced peripheral glucose intolerance. The manuscript is significantly improved after the revision.
