# Supplementary material for: Alzheimer-associated Aβ oligomers impact the central nervous system to induce peripheral metabolic deregulation
Source: EMBO Mol Med. 2015 Jan 23;7(2):190–210. doi: 10.15252/emmm.201404183 (PMC4328648; doi:10.15252/emmm.201404183)
Supplement: Supplementary file 3 [file emmm0007-0190-sd3.pdf]

Clarke et al. - Source Data for Fig. 2F

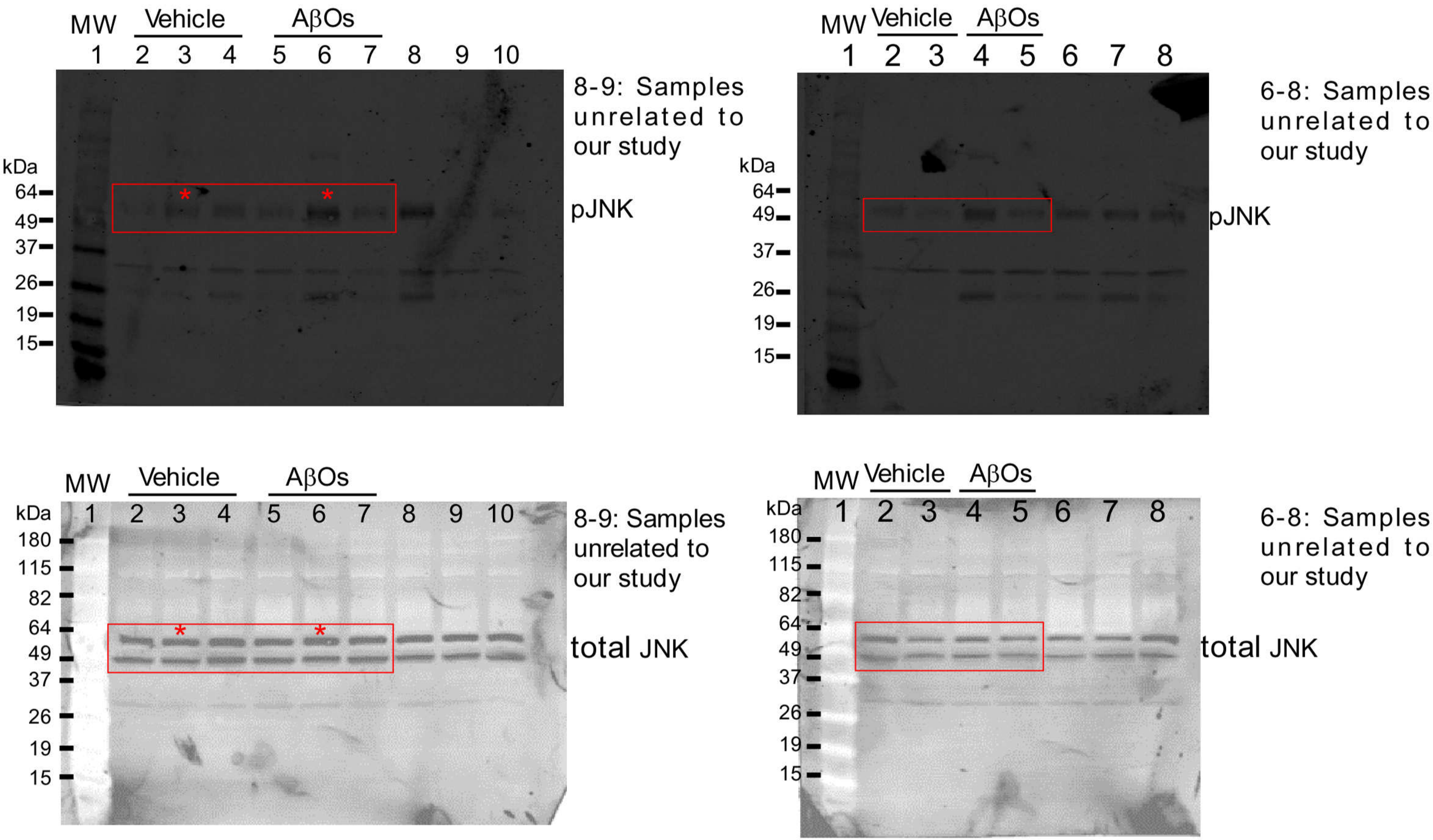

*Representative bands shown in main figure.

Clarke et al. - Source Data for Fig. 2G

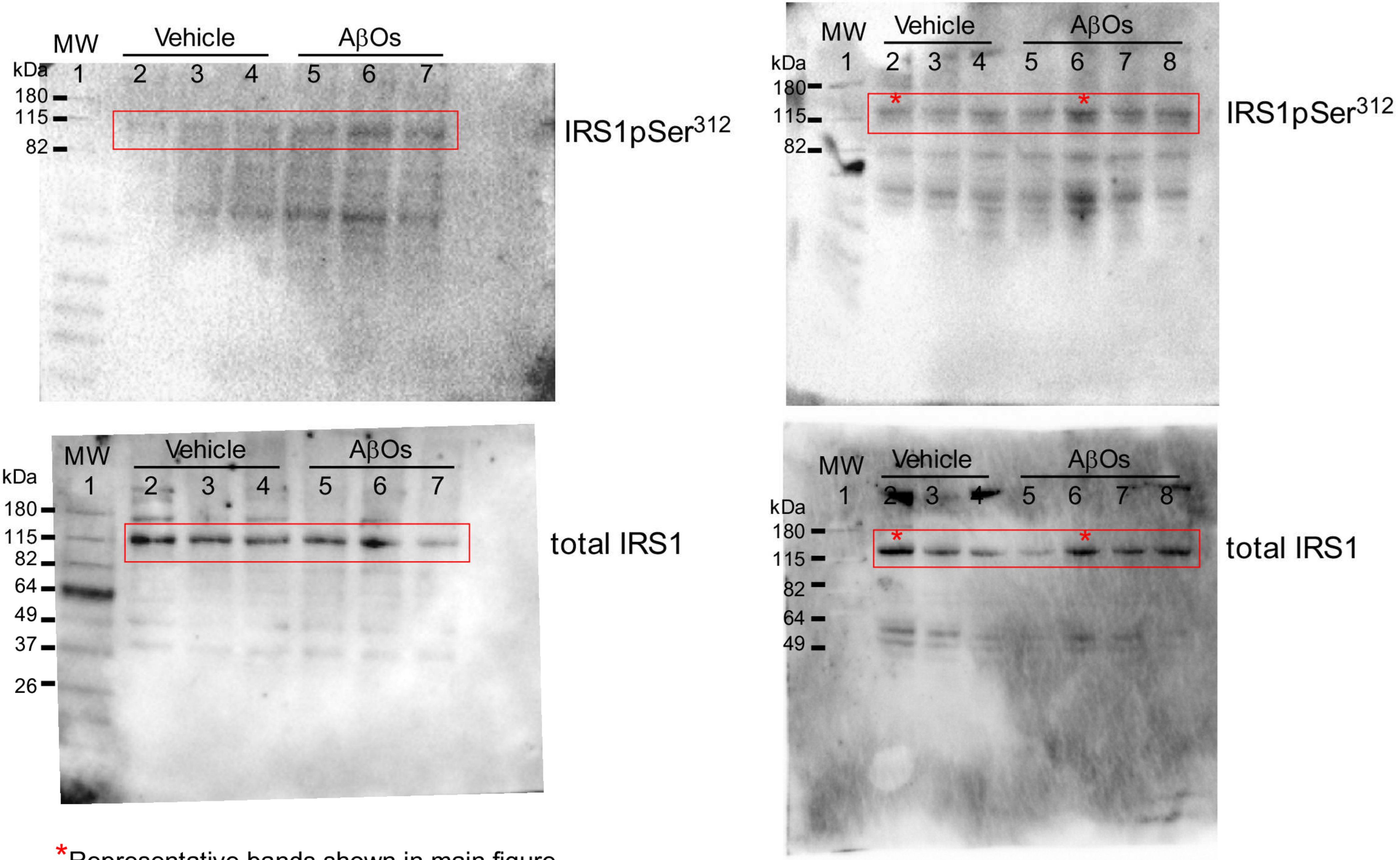

*Representative bands shown in main figure.

Clarke et al. - Source Data for Fig. 2J

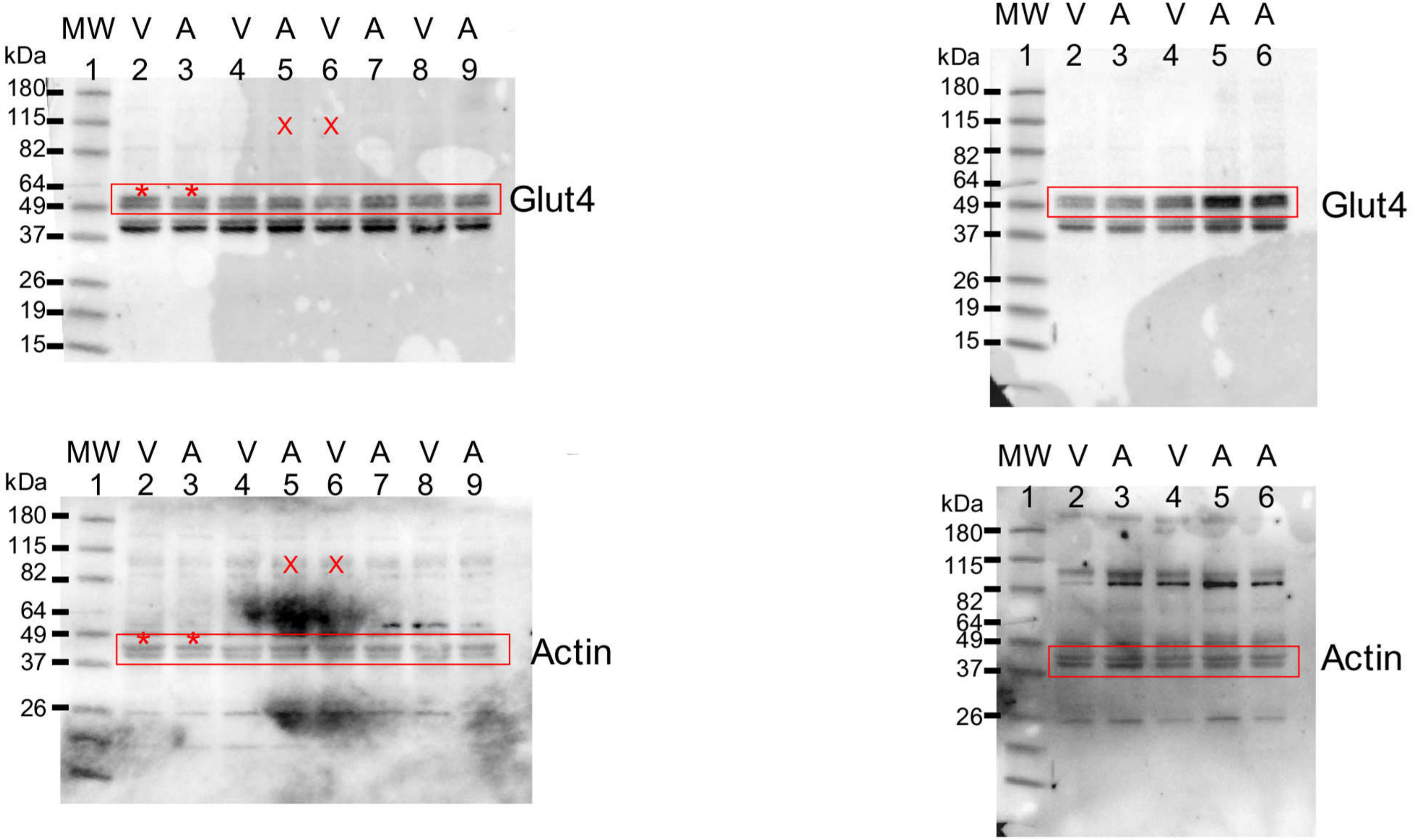

*Representative bands shown in main figure. V = vehicle, A = AβOs

x Lanes not used for quantification.
