# Supplementary material for: Alzheimer-associated Aβ oligomers impact the central nervous system to induce peripheral metabolic deregulation
Source: EMBO Mol Med. 2015 Jan 23;7(2):190–210. doi: 10.15252/emmm.201404183 (PMC4328648; doi:10.15252/emmm.201404183)
Supplement: Supplementary file 4 [file emmm0007-0190-sd4.pdf]

Clarke et al. - Source Data for Fig. 4A

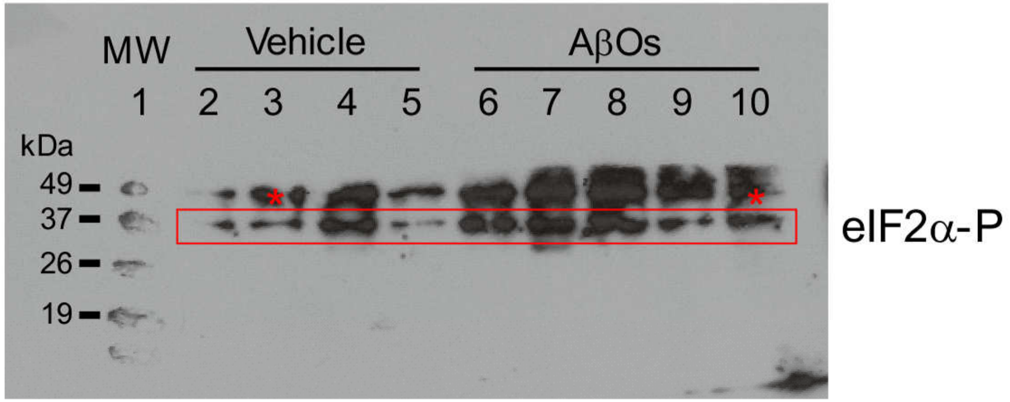

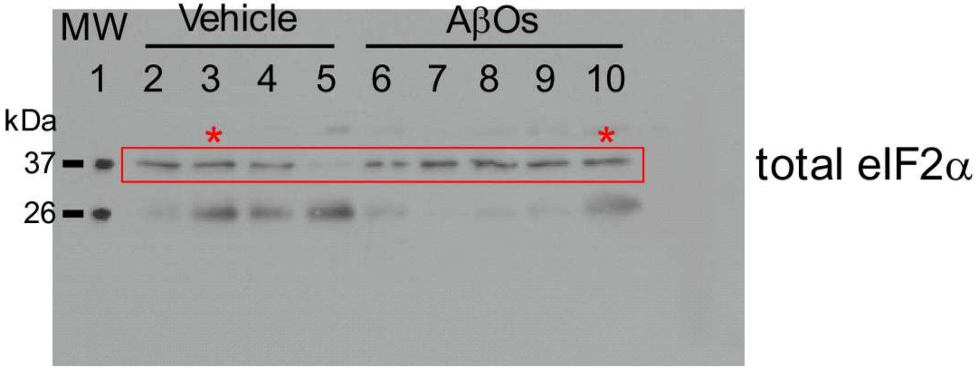

*Representative bands shown in main figure.

Clarke et al. - Source Data for Fig. 4B

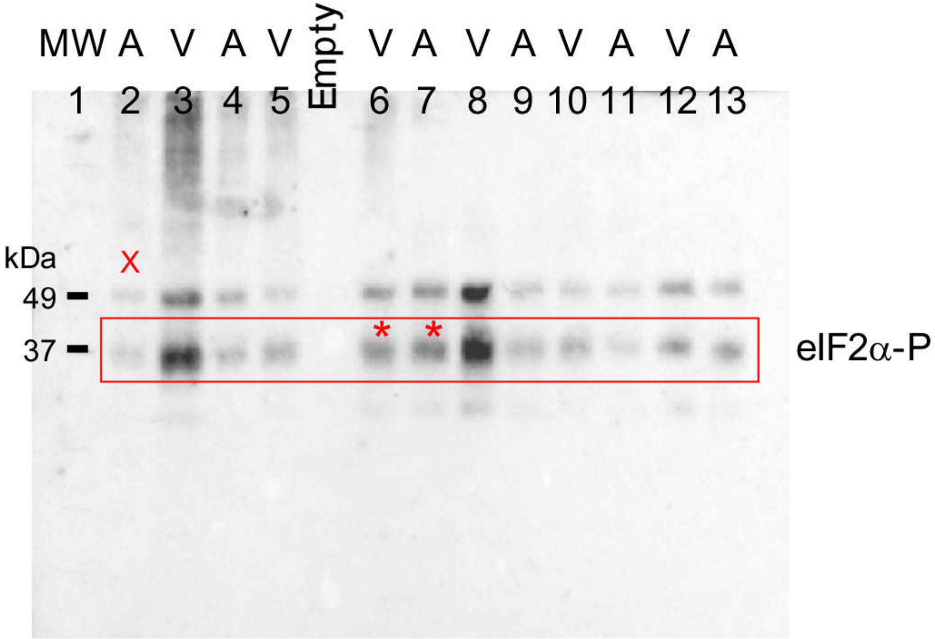

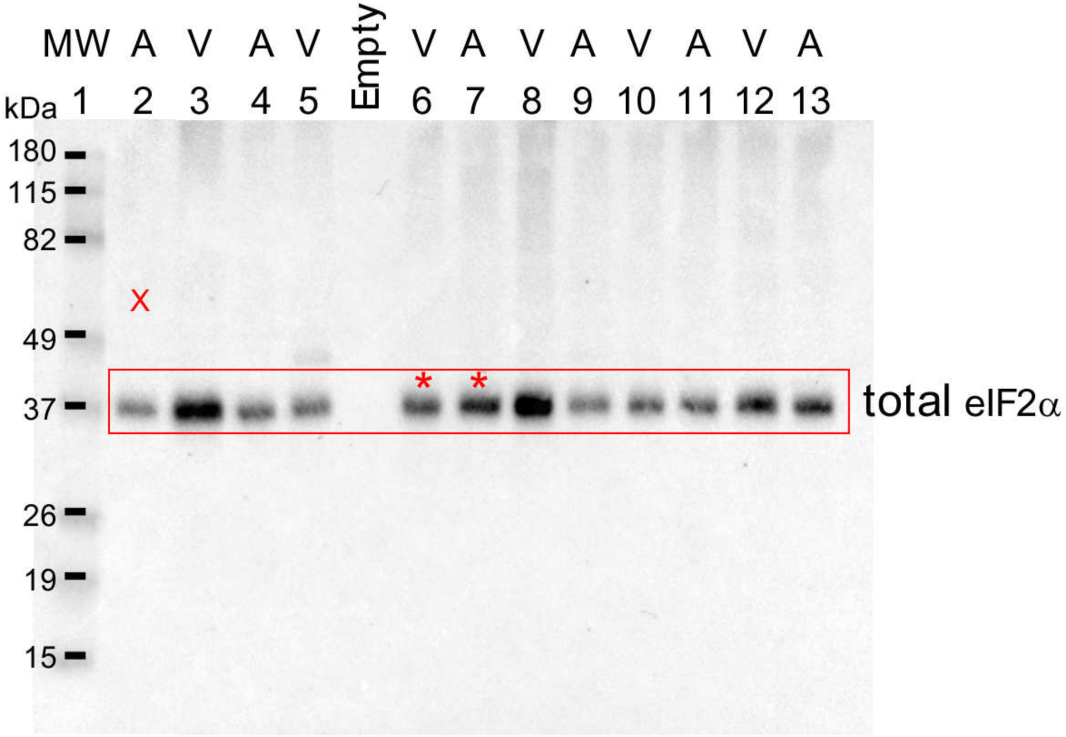

*Representative bands shown in main figure. V = vehicle, A = AβOs

x Lane not used for quantification.

Clarke et al. - Source Data for Fig.4C

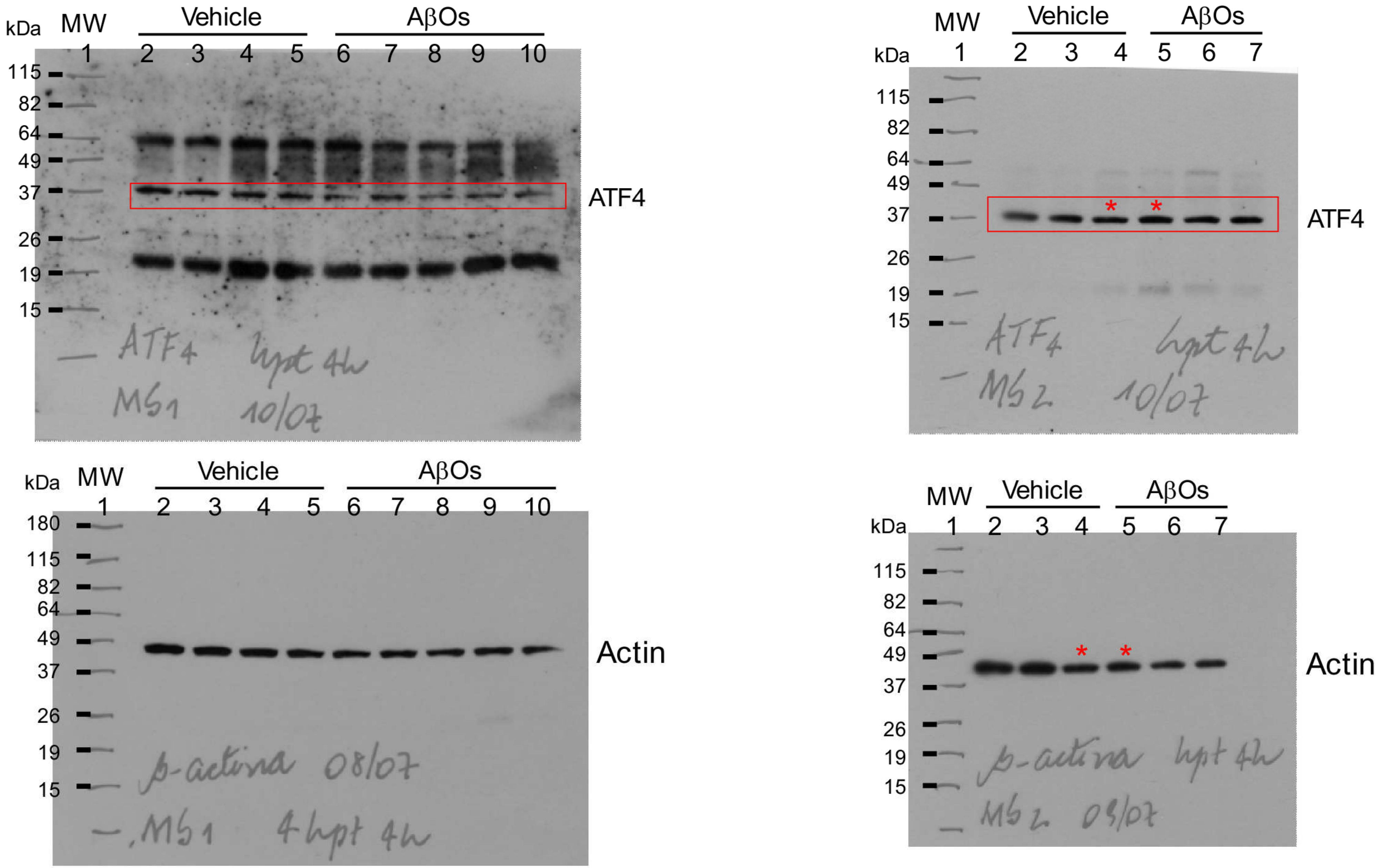

*Representative bands shown in main figure.

Clarke et al. - Source Data for Fig. 4D

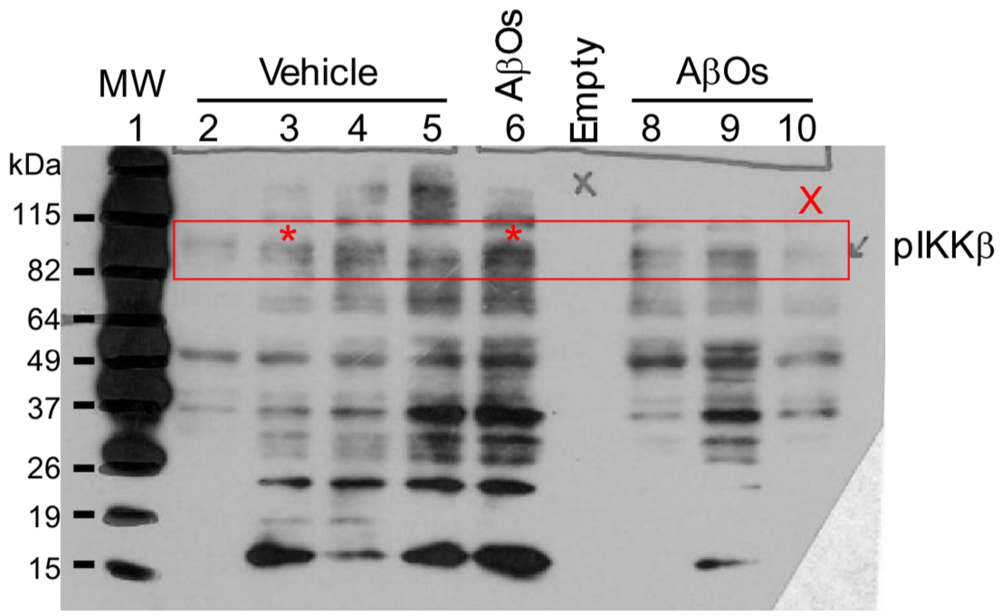

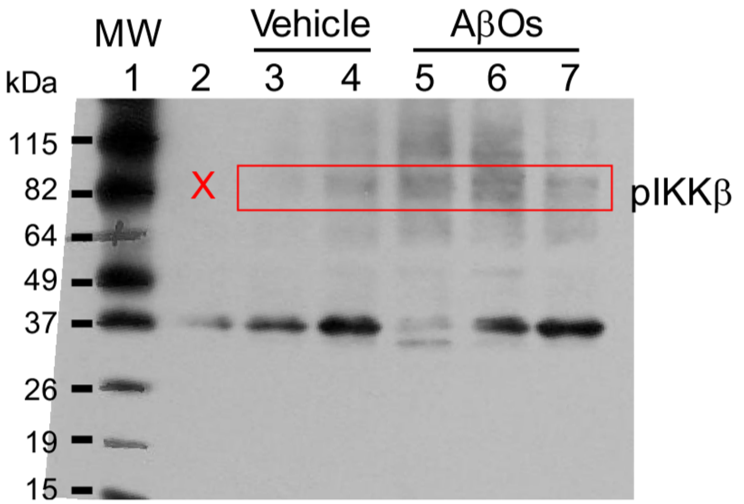

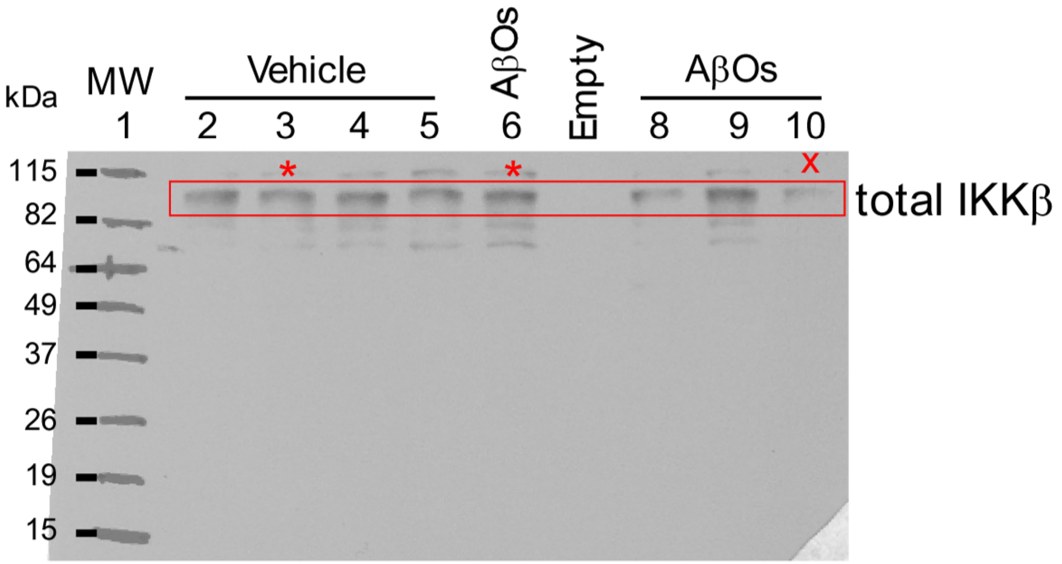

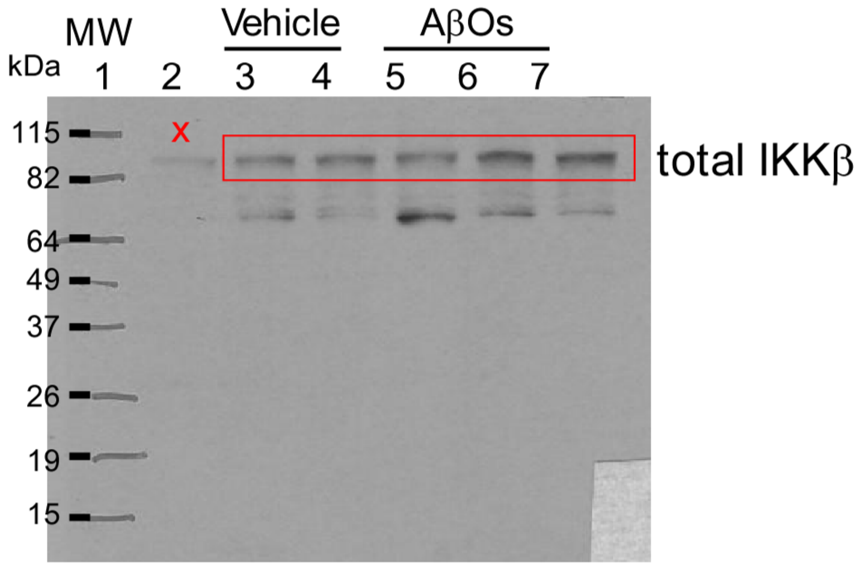

*Representative bands shown in main figure.

x Lanes not used for quantification.

Clarke et al. - Source Data for Fig. 4E

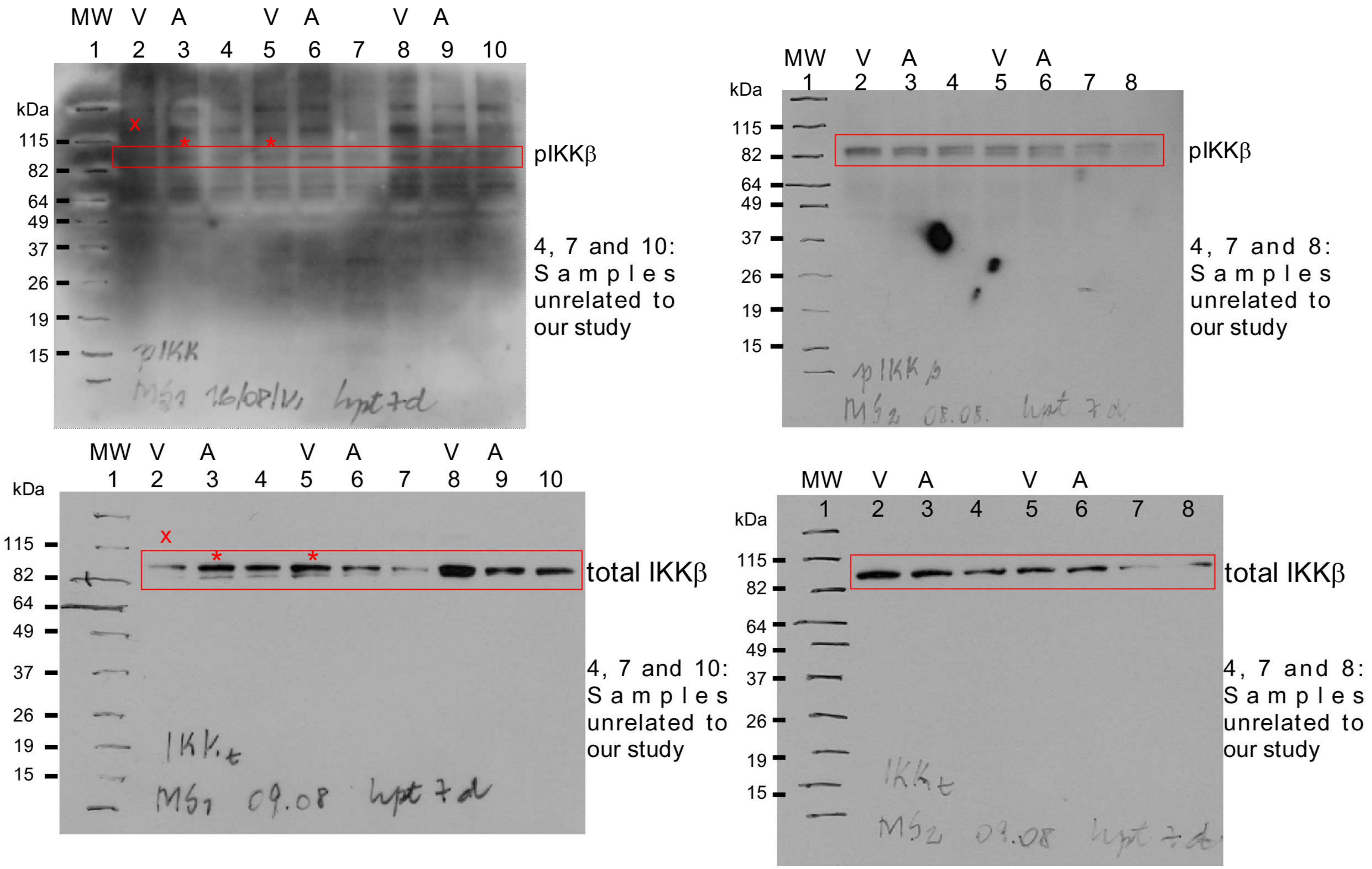

*Representative bands shown in main figure. V = vehicle, A = AβOs

x Lanes not used for quantification.

Clarke et al. - Source Data for Fig. 4F

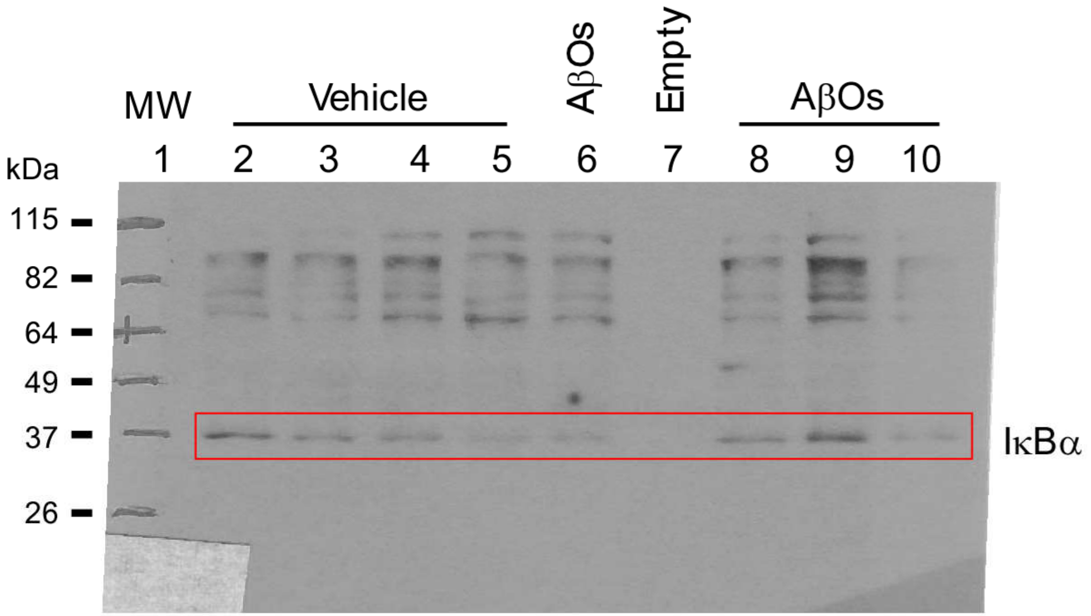

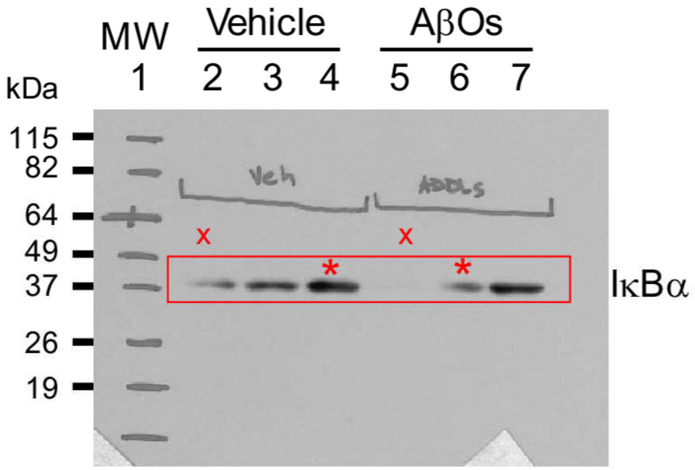

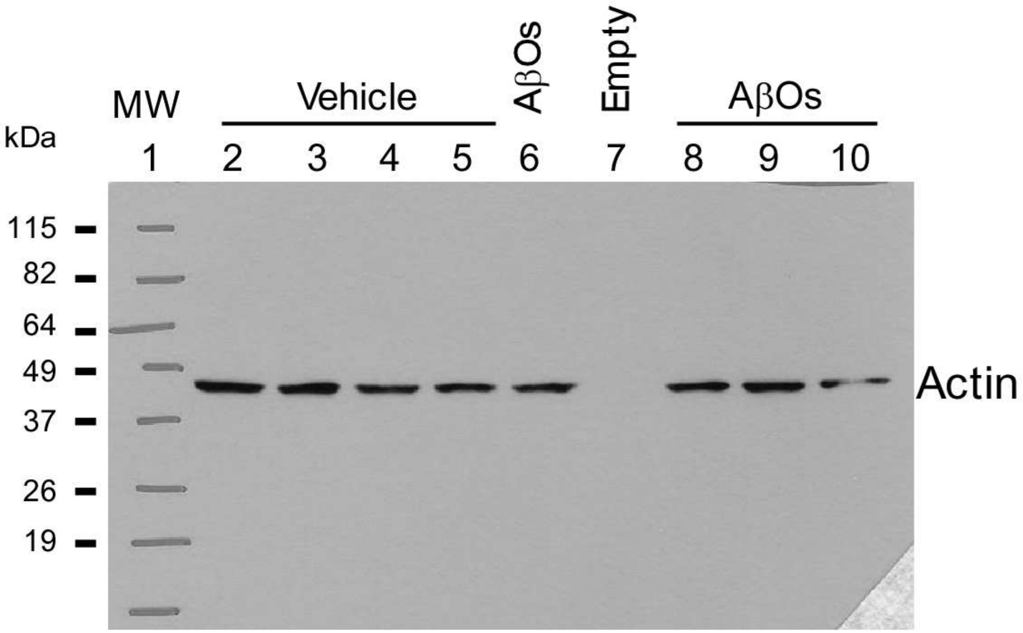

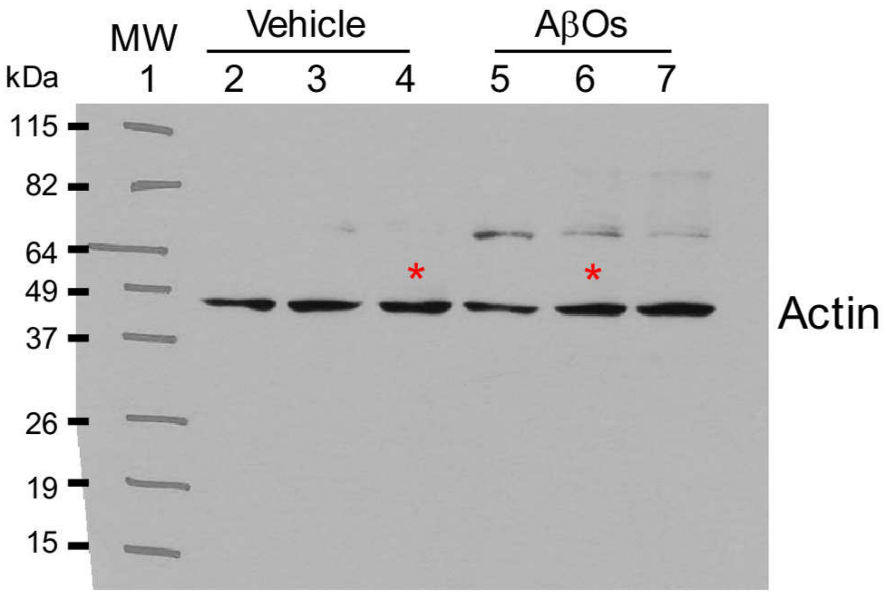

*Representative bands shown in main figure.

x Lanes not used for quantification.

Clarke et al. - Source Data for Fig. 4G

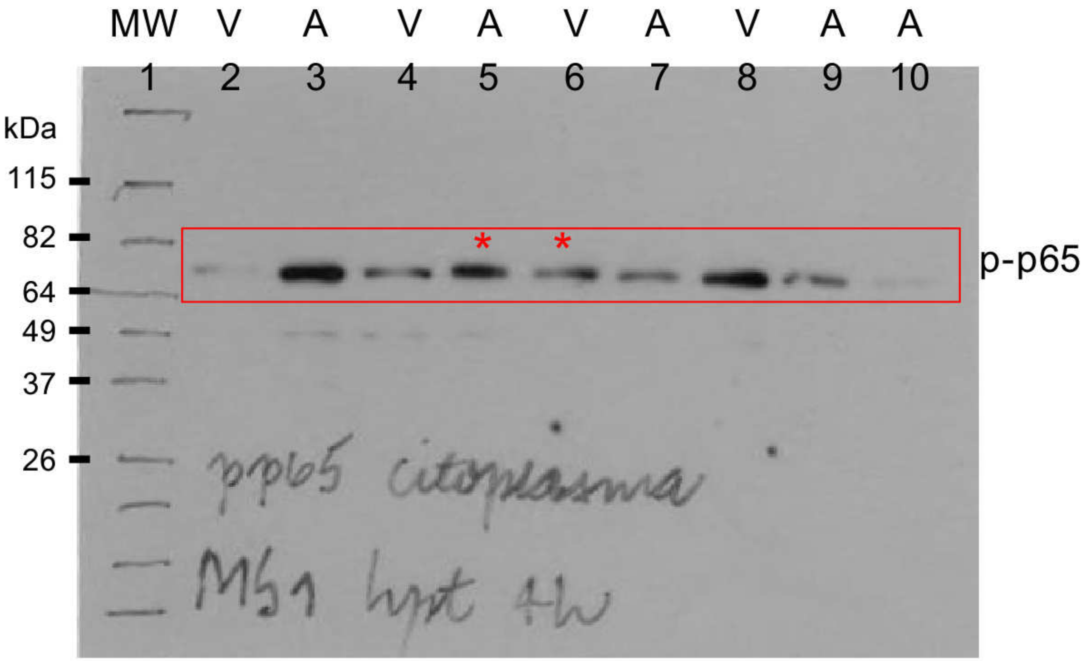

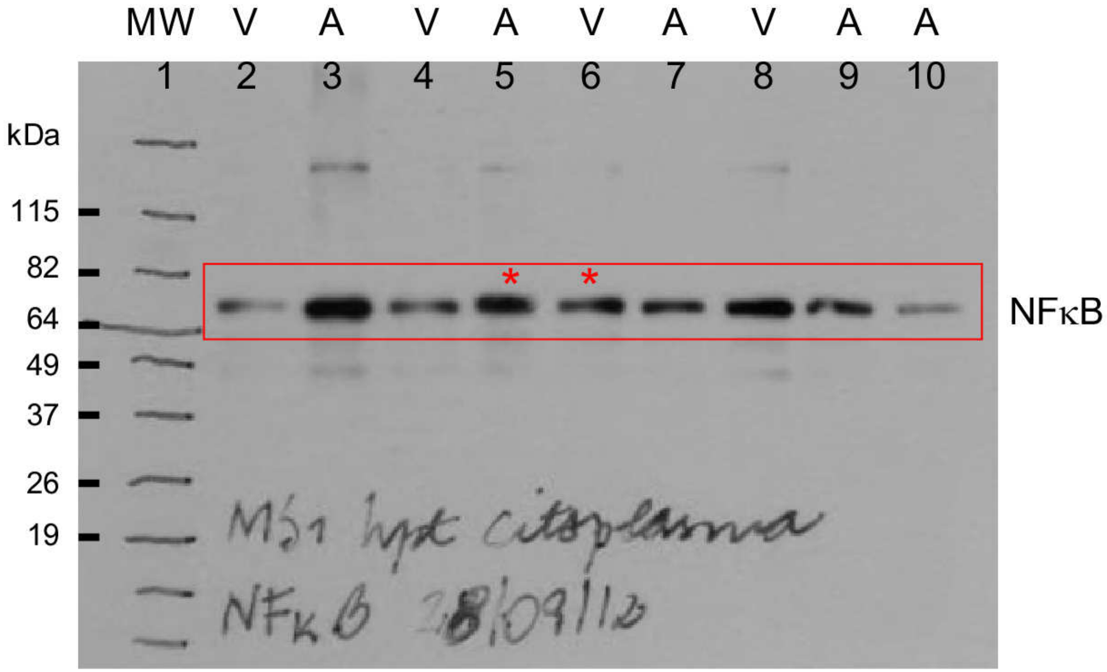

*Representative bands shown in main figure. V = vehicle, A = AβOs

Clarke et al. - Source Data for Fig.4H

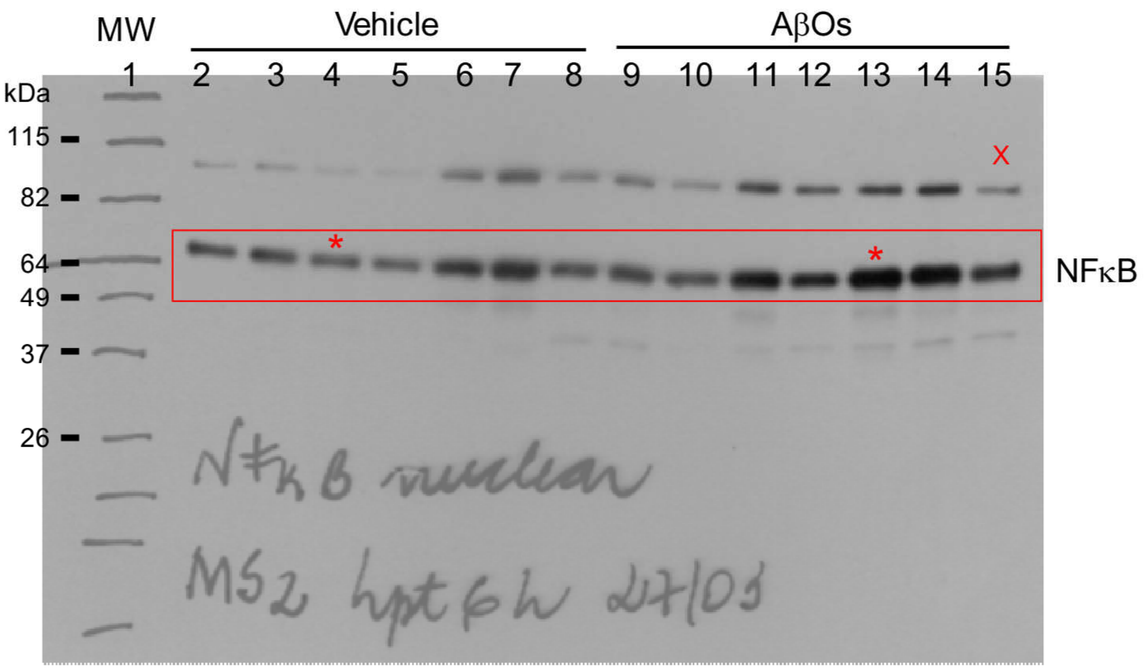

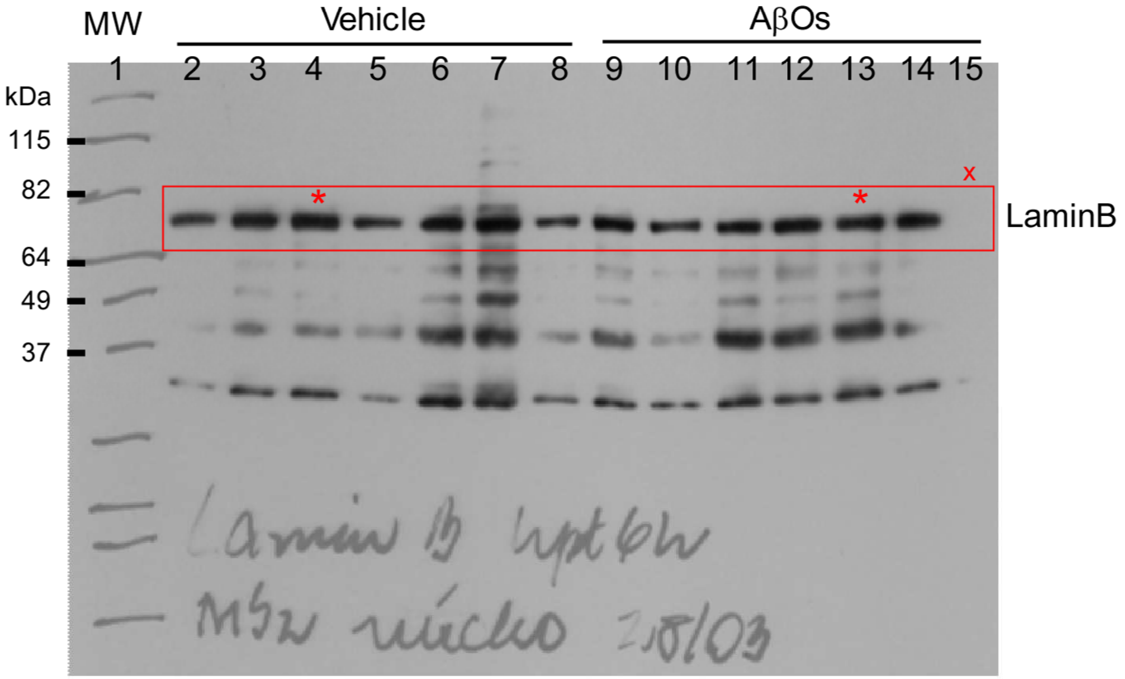

*Representative bands shown in main figure.

x Lanes not used for quantification.

Clarke et al. - Source Data for Fig.4I

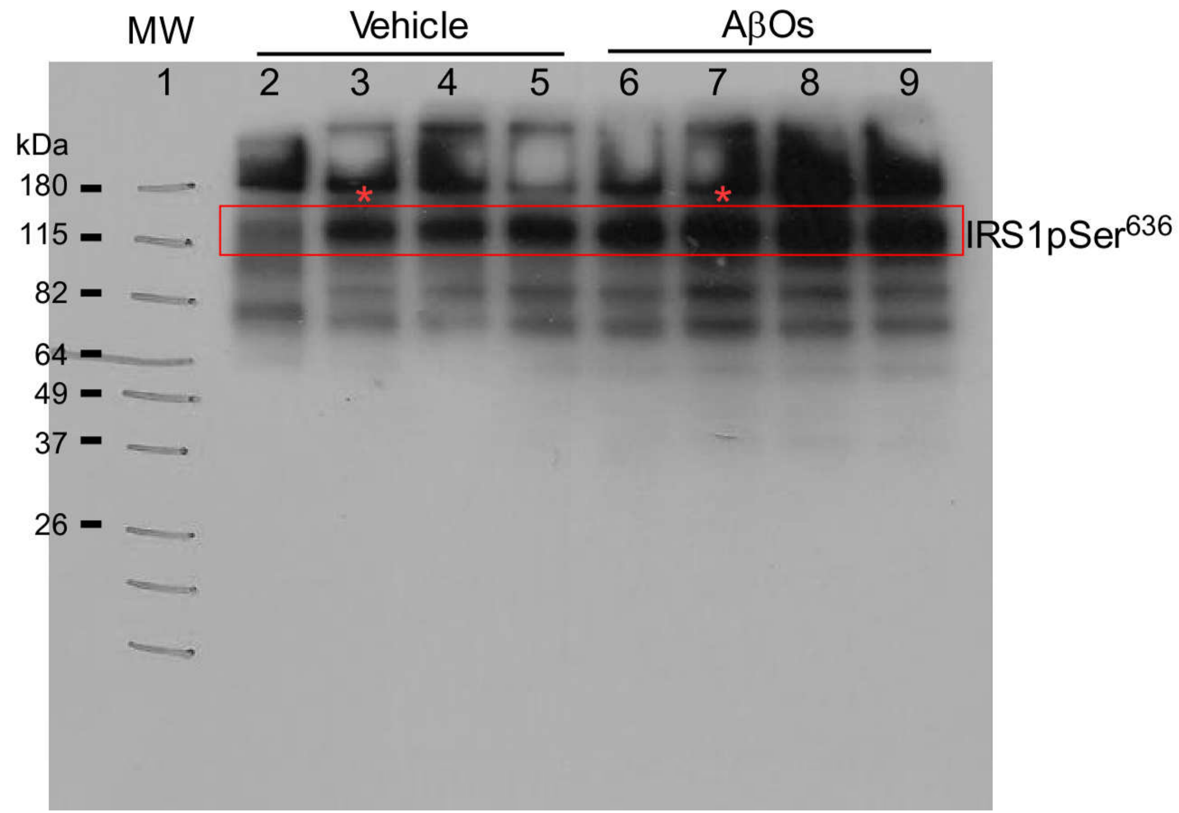

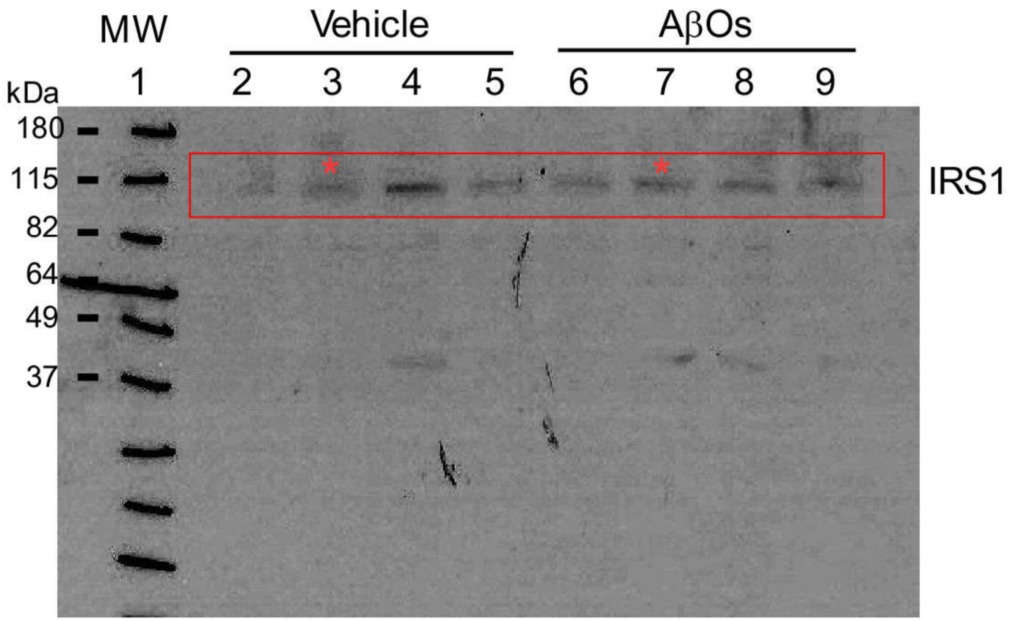

*Representative bands shown in main figure.

Clarke et al. - Source Data for Fig. 4J

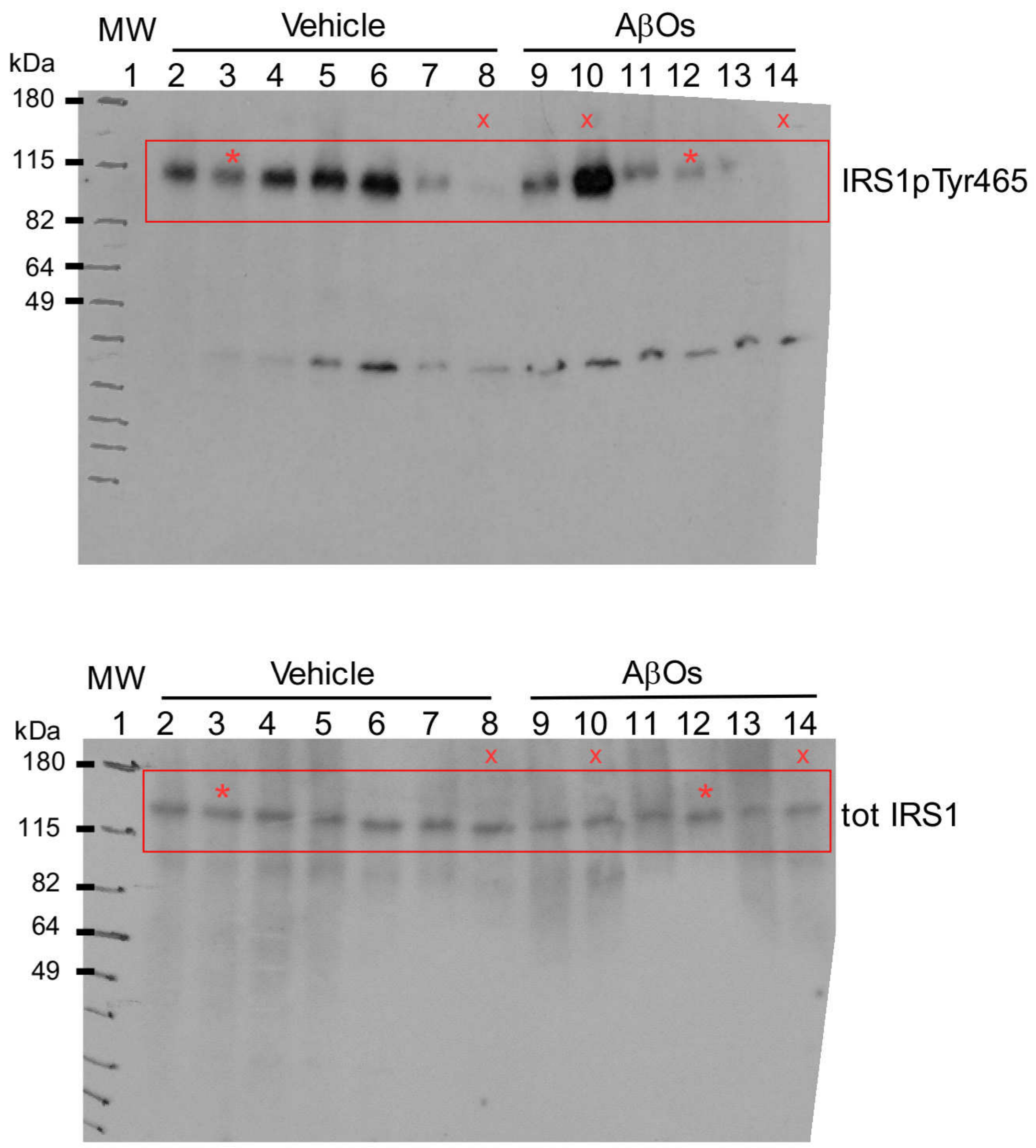

*Representative bands in main figure.
x Lanes not used for quantification.
